# Reconstructing reef fish communities using fish otoliths in coral reef sediments

**DOI:** 10.1371/journal.pone.0218413

**Published:** 2019-06-14

**Authors:** Chien-Hsiang Lin, Brigida De Gracia, Michele E. R. Pierotti, Allen H. Andrews, Katie Griswold, Aaron O’Dea

**Affiliations:** 1 Center for Ecology and Environment, Tunghai University, Taichung, Taiwan; 2 Department of Life Science, Tunghai University, Taichung, Taiwan; 3 Smithsonian Tropical Research Institute, Balboa, Republic of Panama; 4 Department of Oceanography, University of Hawaii at Manoa, HI, United States of America; Uniwersytet Warszawski, POLAND

## Abstract

Little is known about long-term changes in coral reef fish communities. Here we present a new technique that leverages fish otoliths in reef sediments to reconstruct coral reef fish communities. We found over 5,400 otoliths in 169 modern and mid-Holocene bulk samples from Caribbean Panama and Dominican Republic mid-Holocene and modern reefs, demonstrating otoliths are abundant in reef sediments. With a specially-built reference collection, we were able to assign over 4,400 otoliths to one of 56 taxa (35 families) though mostly at genus and family level. Many otoliths were from juvenile fishes for which identification is challenging. Richness (by rarefaction) of otolith assemblages was slightly higher in modern than mid-Holocene reefs, but further analyses are required to elucidate the underlying causes. We compared the living fish communities, sampled using icthyocide, with the sediment otolith assemblages on four reefs finding the otolith assemblages faithfully capture the general composition of the living fish communities. Radiocarbon dating performed directly on the otoliths suggests that relatively little mixing of sediment layers particularly on actively accreting branching coral reefs. All otolith assemblages were strongly dominated by small, fast-turnover fish taxa and juvenile individuals, and our exploration on taxonomy, functional ecology and taphonomy lead us to the conclusion that intense predation is likely the most important process for otolith accumulation in reef sediments. We conclude that otolith assemblages in modern and fossil reef sediments can provide a powerful tool to explore ecological changes in reef fish communities over time and space.

## Introduction

Coral reefs are changing because of local and global human impacts [1–3]. The fossil record can provide important context to these changes by reconstructing pre-human baseline conditions [4,5], revealing underlying mechanisms of change [6], providing accurate conservation objectives [5,7,8], and help predict future changes [9–11]. This “Conservation Paleobiology” approach has been successfully applied to the corals themselves [1,12], but similar efforts for other important members of reef ecosystems lag behind. Historical changes in tropical reef fish communities, for example, are poorly documented, even though they represent a diverse [13] and ecologically-important component of coral reefs [14–16], and have experienced recent drastic changes driven by direct and indirect anthropogenic impacts [17–20].

Coral reef fishes generally have a poor fossil record [21], but in Holocene reef carbonates fish teeth have been shown to be abundant enough to document changes in reef fish communities through millennia [6]. Here we explore a new approach to reconstruct changes in reef fish communities using fish otoliths that accumulate in reef sediments. Otoliths, or fish ear bones, are calcified structures used for balance and hearing in fishes [22]. Until now, it was widely considered that otoliths were rare in carbonate reef sediments to allow meaningful scientific inference due to carbonate cementation and dissolution of aragonite [21,23,24], despite their high abundance and nearly ubiquitous occurrences in other marine soft-bottom sediments [25,26] and their high preservation potential [27]. In this study, we show how careful examination of carbonate sands down to 500 μm in size in unconsolidated reef sediments can yield diverse and abundant otolith assemblages, and these can be used to explore questions related to reef fish community dynamics over time and space.

To assist in the identification of sediment otoliths, we build an otolith reference collection from Neotropical coastal fishes (173 species, see below). We then provide guidelines for extracting and identifying otoliths from reef sediments, and apply this approach to sub-Recent (modern) and mid-Holocene reef sediments from Bocas del Toro and the Dominican Republic. We test the fidelity between otolith sediment assemblages and the living fish communities on reefs and explore taphonomic controls (i.e., how fish remains are buried and fossilized in the sediments) on otolith assemblages in reefs, including the amount of time-averaging (i.e., mixing of sediment layers) through pre- and post-bomb radiocarbon (^14^C) dating of otoliths. Finally, we use these findings to explore the potential applications of otolith assemblages in fossil and modern sediments to establish baselines and monitor reef fish communities.

## Materials and methods

### Setting and site descriptions

**Fig 1:**
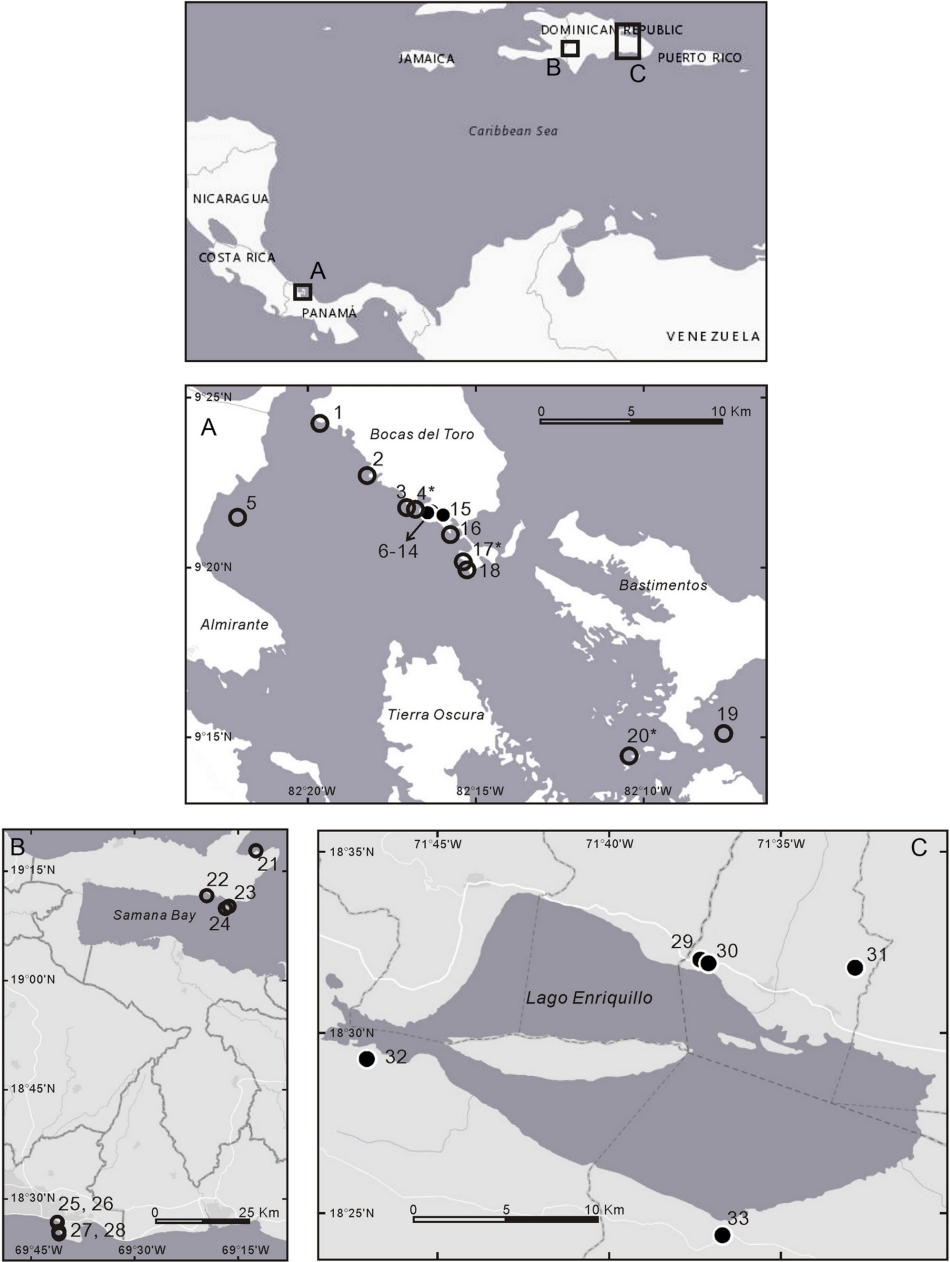
**Sample sites in the Bocas del Toro, Panama (A) and the Dominican Republic (B) (C).** 1. Playa Estrella. 2. Punta Caracol. 3. Casa Blanca. 4. Casa Blanca new. 5. Punta Donato. 6. Sweet Bocas SE Face. 7–14. Sweet Bocas Trench 1–8. 15. Sunset Point. 16. STRI Point. 17. Airport Point. 18. SW Bocas Island. 19. Crawl Cay. 20. Cayo Adriana. 21. La Playita, Las Galeras. 22. Bridge Island reef, Samana Bay. 23. Cayo Levantado, Samana Bay. 24. Feri, SW Levantado, Samana Bay. 25. Las Golondrinas reef, La Caleta. 26. La Caleta Beach Reef. 27. Hickory Ship, La Caleta. 28. Wreck Limon La Caleta. 29. Cañada Honda. 30. Cañon los Rios. 31. Las Clavellinas. 32. Cañon de Buo. 33. Duverge Road Section. Open circle: sub-Recent sites; solid circle: Holocene sites; * indicates rotenone sampling sites. See Tables 1 and 2 for a general description of the sites. (Map was generated by CHL using ESRI, 2018. ArcGIS for Desktop, version 10.5. Environmental Systems Research Institute, Redlands, CA, USA. (http://www.esri.com/)).

**Fig 2:**
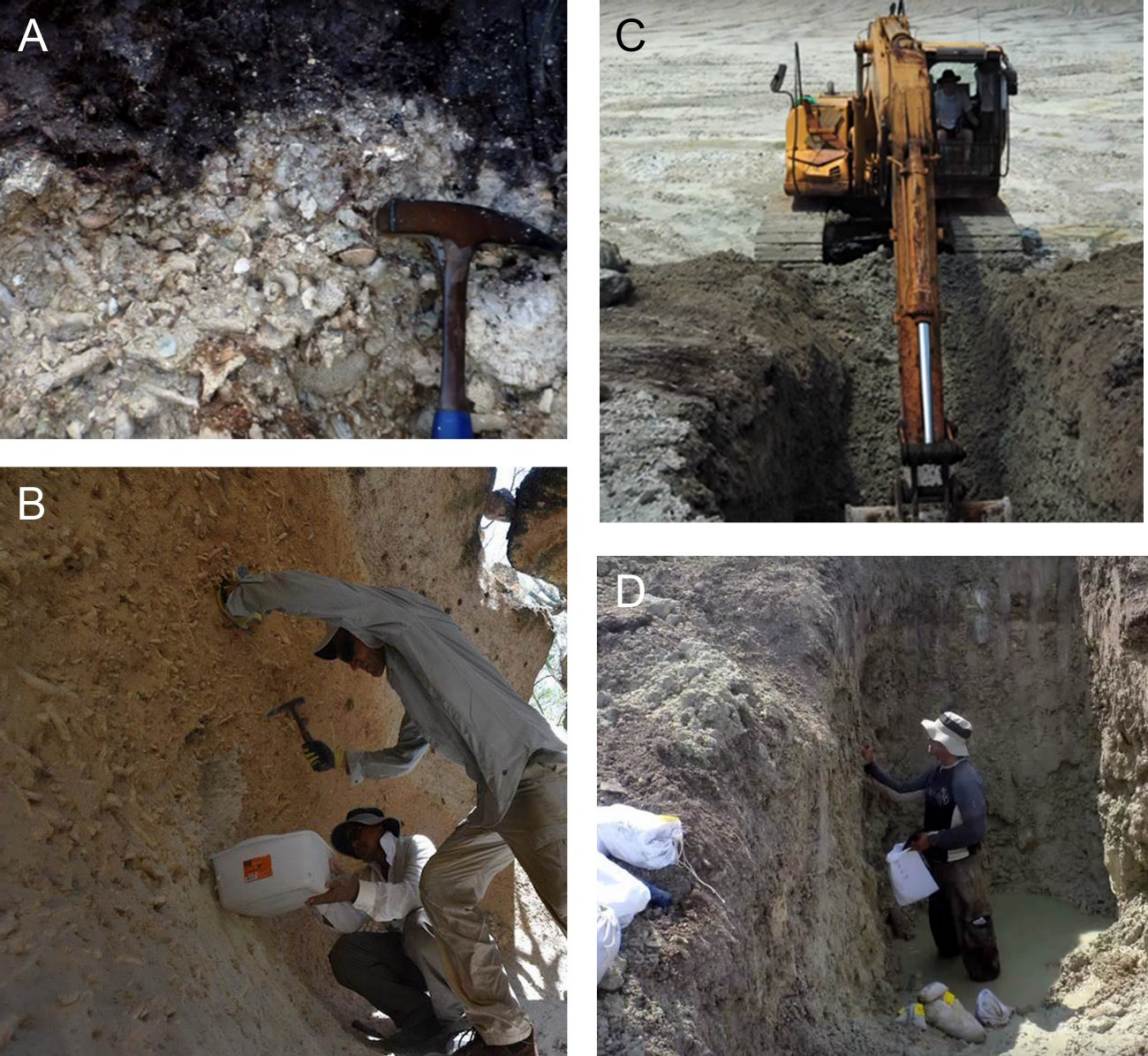
**In situ mid-Holocene fringing coral reefs in Bocas del Toro, western Panama (A, C, D) and the Dominican Republic (B).** Fossil corals are preserved in life position with exquisite preservation. Trenches were dug into the reef and bulk samples of coral communities extracted in Bocas del Toro (**C** and **D**; see also S1 Fig for detailed stratigraphy). Note size of the bulk samples (ca. 1 kg each) is shown in **(D)**.

To explore the use of sediment otolith assemblages we studied two regions (Western Caribbean Panama and the Dominican Republic) where both mid-Holocene and sub-Recent reefs were available for study (Table 1). In Bocas del Toro, western Panama (Fig 1A) a seven-hectare excavation called “Sweet Bocas” revealed a suite of exceptionally well-preserved mid-Holocene fringing coral reefs in Almirante Bay (Fig 2). *In situ* and in life-position corals, including fragile branching corals were preserved in unconsolidated carbonate silts and muds as an autochthonous assemblage (Fig 2A) that accumulated as sea level reached modern-day levels in a sheltered area outside the hurricane belt. The types of habitats exposed at this site include mangrove, seagrass, fringing reef crest, and deeper inter-reef muds, all of which were well-preserved and well-delineated (S1 Fig) [28]. We excavated eight > 3 m-deep trenches (Fig 2C and 2D) and extracted 34 bulk samples roughly 1 kg each in weight. All samples used here came from the reef framework (dominated by *Acropora cervicornis* and *Porites* spp., S1 Fig). These reefs have been dated using U-Th and ^14^C radiometric dating and found to range in age from 7200 to 5700 years BP [28].

**Table 1:** Location and general information of sediment sampling sites.

Bocas del Toro	No. of samples	No. of otoliths	No. of taxa	% unidentified otoliths	Depth (m)/habitat	longitude	latitude
**sub-recent**	**63**	**2498**	**36**	**0.14**			
Airport Point	6	264	19	0.26	5–5.4	-82.25611	9.33611
Casa Blanca	8	372	17	0.17	3.1–3.8	-82.28425	9.36242
Casa Blanca new	6	380	15	0.19	2.4–3.9	-82.27989	9.36194
Cayo Adriana	6	191	16	0.14	3.4–3.7	-82.17384	9.24104
Crawl Cay	3	21	6	0.10	8.7–9.2	-82.12683	9.25155
Playa Estrella	4	407	16	0.09	2.0–10.0	-82.32726	9.40410
Punta Caracol	10	215	16	0.19	1.7–1.9	-82.30383	9.37845
Punta Donato	4	114	12	0.13	3.3–3.5	-82.36809	9.35798
STRI Point	8	384	14	0.04	2.0–12.0	-82.26256	9.34945
SW Bocas Island	8	150	13	0.09	2.0–10.0	-82.25418	9.33184
**Holocene**	**61**	**1953**	**30**	**0.21**			
Sweet Bocas SE Face	18	106	9	0.27	Upper reef slope	-82.27117	9.36082
Sweet Bocas Trench 1	4	26	7	0.35	Upper reef slope	-82.27153	9.36023
Sweet Bocas Trench 2	5	41	7	0.37	Upper reef slope	-82.27143	9.35993
Sweet Bocas Trench 3	5	201	7	0.03	-	-82.27085	9.36051
Sweet Bocas Trench 4	3	5	4	0.20	Upper reef slope	-82.27180	9.35998
Sweet Bocas Trench 5	4	21	5	0.38	Upper reef slope	-82.27275	9.35996
Sweet Bocas Trench 6	9	1036	19	0.26	Upper reef slope	-82.27146	9.36086
Sweet Bocas Trench 7	2	14	6	0.14	Upper reef slope	-82.27273	9.35968
Sweet Bocas Trench 8	2	26	7	0.23	Upper reef slope	-82.27365	9.36018
Sunset Point	9	477	21	0.13	Upper reef slope	-82.26600	9.35900
**Dominican Republic**							
**sub-recent**	**31**	**666**	**34**	**0.25**			
Bridge Island reef, Samana Bay	4	63	11	0.17	9.2	-69.32533	19.19344
Cayo Levantado, Samana Bay	4	81	12	0.32	5.1–8.2	-69.27211	19.16965
Feri, SW Levantado, Samana Bay	4	36	10	0.08	7.6–10.2	-69.28076	19.16459
Hickory Ship, La Caleta	4	183	15	0.32	12.9–17.2	-69.68323	18.42611
La Caleta Beach Reef	4	146	14	0.19	10.8–11.9	-69.68654	18.44639
La Playita, Las Galeras	3	19	6	0.42	10.8–11.2	-69.20724	19.29641
Las Golondrinas reef, La Caleta	4	34	8	0.29	11.2–13.1	-69.68836	18.44947
Wreck Limon La Caleta	4	104	14	0.21	16.6–19.2	-69.68293	18.42407
**Holocene**	**14**	**296**	**16**	**0.16**			
Cañada Honda	2	27	4	0.30	-	-71.62207	18.53367
Cañon de Buo	3	67	9	0.13	-	-71.78400	18.48800
Cañon los Rios	4	99	6	0.16	Reef core	-71.61820	18.53200
Duverge Road Section	1	16	4	0.00	Upper reef slope	-71.61140	18.40665
Las Clavellinas	4	87	10	0.17	Upper reef slope	-71.54700	18.53005

In the Dominican Republic, we sampled similar-aged mid-Holocene reefs exposed in a number of storm channels located around the Enriquillo Basin (Fig 1C) [29]. These fossil reefs have been more intensively studied than the fossil reefs in Panama, and have been previously dated to between ~9400 and 5400 years BP by Greer et al. [30]. Like the Panama fossil reefs, they preserve *in situ* and in-life position corals and the matrix is neither consolidated nor indurated (Fig 2B). For further descriptions of the fossil reefs, see Reuter et al. [31], Stemann and Johnson [32], Cuevas et al. [33], Lescinsky et al. [34], Mann et al. [29], and Greer et al. [30].

In both regions, comparable bulk samples were also taken from modern branching coral reef framework on living reefs (Fig 1A and 1B) by excavating into the reef framework next to living corals to a depth of ~10 cm at water depths of 2–19 m (S2 Fig).

Permits for collecting bulk sediment samples of both sub-Recent and mid-Holocene in Panama and Dominican Republic were issued by the Ministerio de Ambiente, Republica de Panamá (Permit number SE/AO-4-18) and the Ministerio de Medio Ambiente y Recursos Naturales, República Dominicana (Permit number VAPB-02374), respectively.

### Extraction, identification, and analysis of otoliths in sediments

The bulk samples of sub-Recent and mid-Holocene reef matrix were dried and weighed, then wet-sieved over 2 mm, 500 μm, 250 μm, and 106 μm-mesh screens. Sediment in the 2 mm and 500 μm fractions was carefully scanned under a dissecting microscope and all otoliths picked from the carbonate sands. Each isolated otolith was identified using regional literature [35–38], established databases (https://otolithspisciumpanama.jimdo.com/) [39] and our specially-built in-house reference collection (see below). Identifications made to the highest taxonomic resolution confidently possible, although all identifications conservative. Most specimens were recognised to family or genus. A small proportion of otoliths could not be confidently identified even to family. In some rare cases, otolith specimens could be assigned to species, but only when they were well-preserved and our reference collection was complete for that group. Assignment of some important and rare taxa deserves further explanations, for the purpose of future replication they are highlighted in the Systematics section. The otoliths extracted from the sediment samples are available at the Naos Marine Laboratories of the Smithsonian Tropical Research Institute (STRI), Panama under the category “otolith” without specimen numbers. All necessary permits (see above) were obtained for the described study, which complied with all relevant regulations.

The abundance of otoliths in each taxon in each sample was counted. Other fish remains such as spines, teeth, and vertebrae can be found sporadically from the residues as well, but were much rarer in the > 500 μm fraction. These were kept but are not further discussed here. Average otolith density in each sample was computed as otolith count/dry sediment weight (kg). Richness and diversity estimates were calculated per sample and sample-based rarefaction and extrapolation with 95% confidence intervals calculated using EstimateS software [40]. Further details of the statistics used can be found in S1 Text.

### Testing the fidelity of otolith assemblages in reefs

To explore how faithful otolith sediment assemblages represent living fish communities we conducted small rotenone surveys on four reefs in Bocas del Toro and compared the survey results to our otolith assemblages from the sub-Recent bulk samples on the same reefs (Fig 1A). These sites were previously found to be rich in sediment otoliths and the corresponding rotenone stations were taken close to the sediment sampling locations. A total of eight rotenone stations (each reef with two replicates) were conducted (Table 2). Permit for collecting fish samples using rotenone in Panama was issued by the Ministerio de Ambiente, Republica de Panamá (Permit number SC/A-18-18). This study was carried out in strict accordance with the recommendations in the Collaborative Institutional Training Initiative (CITI) Program course for working with fish in research settings (Record ID: 28175614). The protocol was approved by the Committee on the Ethics of Animal Experiments of Smithsonian Institution.

**Table 2:** Location and general information of rotenone sampling sites.

Rotenone sampling	No. of samples	No. of individuals	No. of taxa	Depth (m)	longitude	latitude
Airport Point (shallow)	2	42	11	1.7–1.9	-82.25611	9.33611
Airport Point (deep)	2	207	16	5.5–6.1	-82.25611	9.33611
Cayo Adriana	2	36	7	4.1–5.9	-82.17384	9.24104
Casa Blanca new	2	111	13	2.5–4.3	-82.27989	9.36194
total	8	396	26			

Rotenone is the most effective tool for reef fish diversity surveys in small areas [41–44] and is especially useful in this case because the approach enables the inclusion of the otherwise hard to sample cryptobenthic fish fauna. An aqueous rotenone mixture was prepared following Robertson and Smith-Vaniz [44] and applied an enclosed blocknet that surrounded approximately 1.8 m^2^ of the reef at each station [42,43]. All fishes extracted after treatment with rotenone were identified [45,46], their otoliths removed, and the length of the fish and their otoliths measured.

Sediment otolith assemblages and rotenone survey samples were compared based upon their taxonomic composition and abundance. Rotenone survey samples with less than 10 individuals of fishes were first excluded. Samples from the same site were then grouped together to increase their sample size in subsequent analyses. Higher taxonomic ranks were adopted and aligned (mainly the rotenone samples, to family or genus level) to make sediment otolith assemblages and rotenone assemblages comparable. In addition, fish otoliths that were measured to be less than 500 μm in the rotenone samples were excluded because the sediments were sieved at that mesh size. All anchovies (Engraulidae), herrings (Clupeidae), codlets (Bregmacerotidae), and silversides (Atherinidae and Atherinopsidae) were excluded from the sediment assemblages because the rotenone survey technique fails to capture mobile, epipelagic fishes [44]. Similarities between sediment otolith assemblages and rotenone survey samples were assessed using a paired group cluster analysis and non-metric multidimensional scaling (nMDS) with Bray-Curtis similarity index based upon their taxonomic composition and relative abundances.

### Exploring time averaging in modern otolith assemblages

Uranium-Thorium (U-Th) dates of coral skeleton show that coral pieces in branching reef framework undergo limited post-burial vertical mixing [6], presumably because they create a framework resistant to bioturbation and storm action. However, smaller skeletal elements such as otoliths that can be as small as 500 μm in size and less than 0.5 mg in mass could be more susceptible to vertical mixing. The problem is that dating such small elements is not straightforward. U-Th dating is not applicable as otoliths do not uptake U when the fish is alive. Amino-acid racemization requires considerably larger sample sizes and calibration with standard radiocarbon dating [47].

Standard radiocarbon dating can be applied to small otolith masses but are subject to marine reservoir age (an uncertain depletion of the radiocarbon pool due to the unknown age of environmental waters at the time of formation), which can reduce temporal resolution. However, dates after 1950—the period of atmospheric testing of nuclear bombs—provides a sharp increase of environmental ^14^C that can be used in determining time averaging from recent sediments. In this case, both the rise of atmospheric ^14^C and the post-peak decline can be used as a chronometer when properly analysed [48].

We, therefore, used bomb pulse dating to explore the ages of the small otoliths in modern seafloor assemblages. From two bulk samples, we randomly selected 20 Gobiidae otoliths, within mass ranges 0.56–0.89 mg (S1 Table). Otoliths were submitted as carbonate to the National Ocean Sciences Accelerator Mass Spectrometry Facility (NOSAMS), Woods Hole Oceanographic Institution (Woods Hole, Massachusetts) for standard hydrolysis analyses to determine sample ^14^C levels. Radiocarbon measurements were reported by NOSAMS as Fraction Modern—the measured deviation of the ^14^C/^12^C ratio from Modern. Modern is defined as 95% of the ^14^C concentration of the National Bureau of Standards Oxalic Acid I standard (SRM 4990B) normalised to δ^13^CVPDB (–19‰) in 1950 AD [49]. Radiocarbon results were corrected for isotopic fractionation using δ^13^C measured concurrently during AMS analysis and are reported here as F^14^C [50]. Further details of the carbon dating method can be found in S2 Text.

## Otolith reference collection

Comparative systematics is critical for the correct identification of isolated otoliths [23]. Regional otolith monographs and databases have been published worldwide to facilitate their identification [23], but otoliths from Caribbean reefs have received no systematic attention to the best of our knowledge. The most relevant literature on shore fish otoliths is restricted to Indo-Pacific taxa [51,52], though papers on otoliths of specific group from tropical America exist [53–58]. This is problematic because of the often distant phylogenetic relationships with Caribbean fauna and the absence of Western Atlantic fish families in the Indo-Pacific. In response, we generated and curated a reference otolith collection of Neotropical reef-associated fishes. Beginning in 2010, specimens were collected by a variety of means including: fish landings from different markets in Tropical America and their bycatch, assistance from local fishermen, donation of fishes from pre-existing local collections, and rotenone collecting in this study (see below, and S3–S6 Figs).

The reference collection includes over 1500 otolith specimens belonging to 173 species of 52 families (S2 Table). Several taxa include representatives from different ontogenetic stages (e.g., S6 Fig). The collection is available for study at the Naos Marine Laboratories of the Smithsonian Tropical Research Institute (STRI) and is continually expanding in geographic, taxonomic and ontogenetic scope.

## Systematics

**Fig 3:**
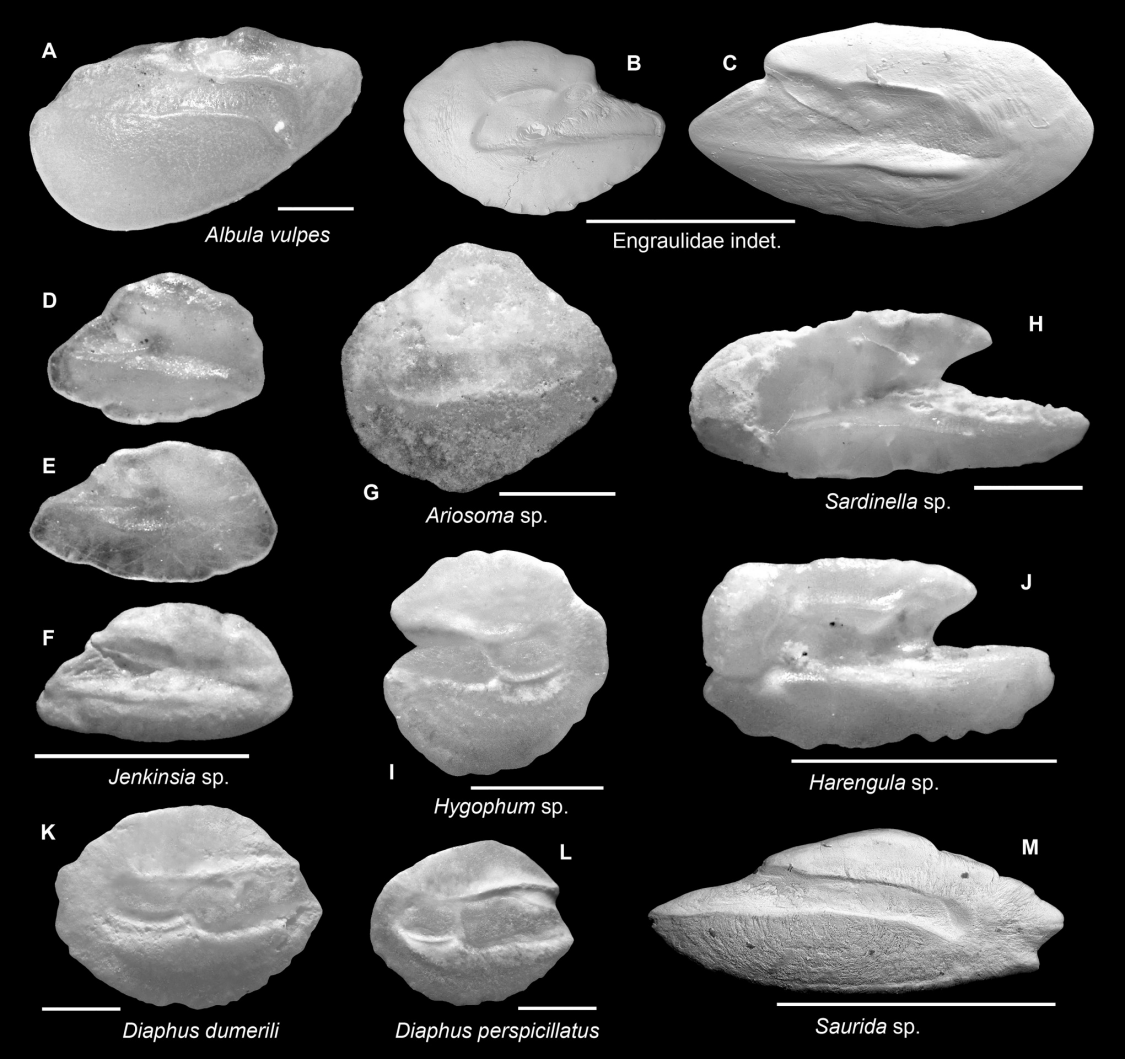
Fish otoliths from the mid-Holocene and sub-Recent reef sediments. Images are inner views and scale bars = 1 mm. **A.** *Albula vulpes*, right otolith, mid-Holocene, Sweet Bocas. **B, C.** Engraulidae indet., mid-Holocene, B, left otolith, Cañon de Buo; C, right otolith, Sunset Point. **D-F.** *Jenkinsia* sp., right otoliths, D, mid-Holocene, Sunset Point; E, sub-Recent, STRI Point; F, sub-Recent, Punta Caracol. **G.** *Ariosoma* sp., right otolith, sub-Recent, Wreck Limon La Caleta. **H.** *Sardinella* sp., left otolith, sub-Recent, Feri, SW Levantado, Samana Bay. **I.** *Hygophum* sp., right otolith, sub-Recent, Hickory Ship La Caleta. **J.** *Harengula* sp., left otolith, mid-Holocene, Sweet Bocas SE Face. **K.** *Diaphus dumerili*, left otolith, sub-Recent, Hickory Ship La Caleta. **L.** *Diaphus perspicillatus*, left otolith, sub-Recent, Hickory Ship La Caleta. **M.** *Saurida* sp., right otolith, sub-Recent, STRI Point.

**Fig 4:**
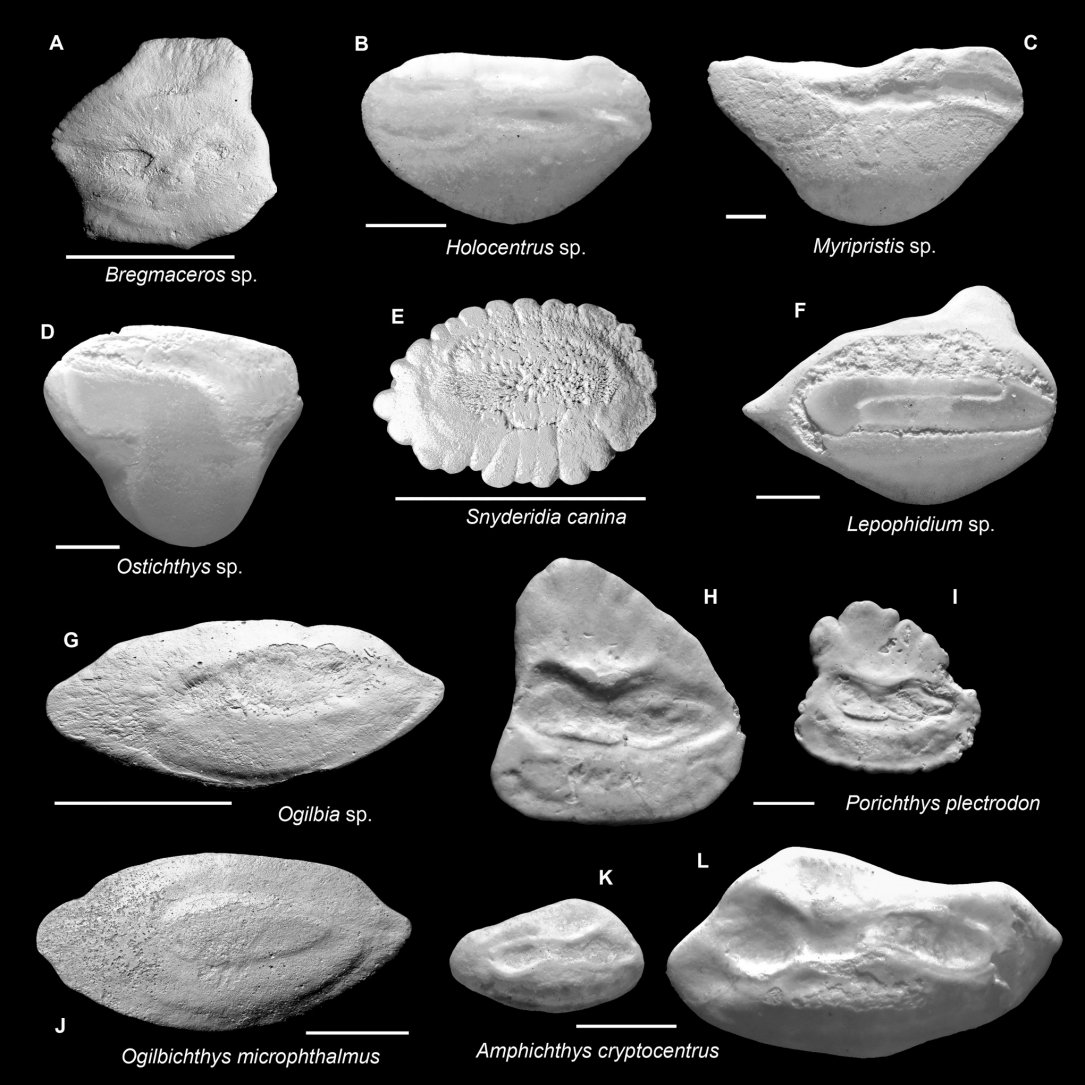
Fish otoliths from the mid-Holocene and sub-Recent reef sediments. Images are inner views and scale bars = 1 mm. **A.** *Bregmaceros* sp., left otolith, mid-Holocene, Cañon de Buo. **B.** *Holocentrus* sp., right otolith, sub-Recent, La Caleta Beach Reef. **C.** *Myripristis* sp., right otolith, sub-Recent, Hickory Ship, La Caleta. **D.** *Ostichthys* sp., right otolith, sub-Recent, Wreck Limon La Caleta. **E.** *Snyderidia canina*, left otolith, sub-Recent, Playa Estrella. **F.** *Lepophidium* sp., left otolith, sub-Recent, Cayo Levantado, Samana Bay. **G.** *Ogilbia* sp., left otolith, sub-Recent, La Caleta Beach Reef. **H, I.** *Porichthys plectrodon*, left otoliths, H, sub-Recent, SW Bocas Island; I, mid-Holocene, Sweet Bocas Trench 8. **J.** *Ogilbichthys microphthalmus*, left otolith, sub-Recent, Hickory Ship La Caleta. **K, L.** *Amphichthys cryptocentrus*, sub-Recent, K, left otolith, Cayo Adriana; L, right otolith, STRI Point.

**Fig 5:**
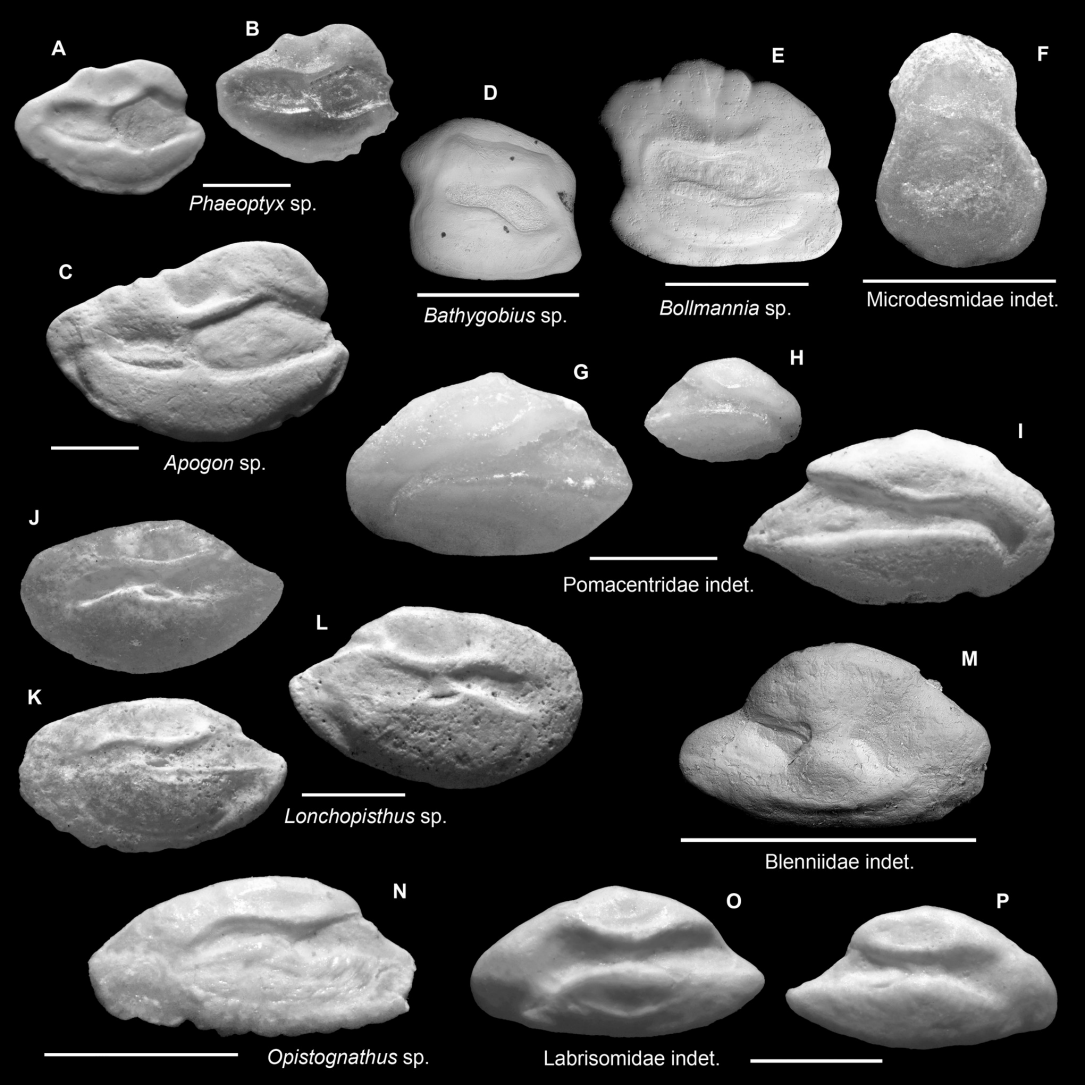
Fish otoliths from the mid-Holocene and sub-Recent reef sediments. Images are inner views and scale bars = 1 mm. **A, B.** *Phaeoptyx* sp., left otoliths, sub-Recent, A, Casa Blanca; B, SW Bocas Island. **C.** *Apogon* sp., left otolith, sub-Recent, SW Bocas Island. **D.** *Bathygobius* sp., left otolith, mid-Holocene, Sunset Point. **E.** *Bollmannia* sp., left otolith, sub-Recent, SW Bocas Island. **F.** Microdesmidae indet., right otolith, mid-Holocene, Cañon de Buo. **G-I.** Pomacentridae indet., G, left otolith; H, I, right otoliths; G, H, mid-Holocene, Sweet Bocas Trench 2; I, sub-Recent, Wreck Limon La Caleta. **J-L.** *Lonchopisthus* sp., J, K, left otoliths; L, right otolith; J, L, sub-Recent, Playa Estrella; K, mid-Holocene, Sweet Bocas Trench 7. **M.** Blenniidae indet., right otolith, mid-Holocene, Las Clavellinas. **N.** *Opistognathus* sp., left otolith, sub-Recent, Casa Blanca. **O, P.** Labrisomidae indet., sub-Recent, O, left otolith, Hickory Ship, La Caleta; P, right otolith, Wreck Limon La Caleta.

**Fig 6:**
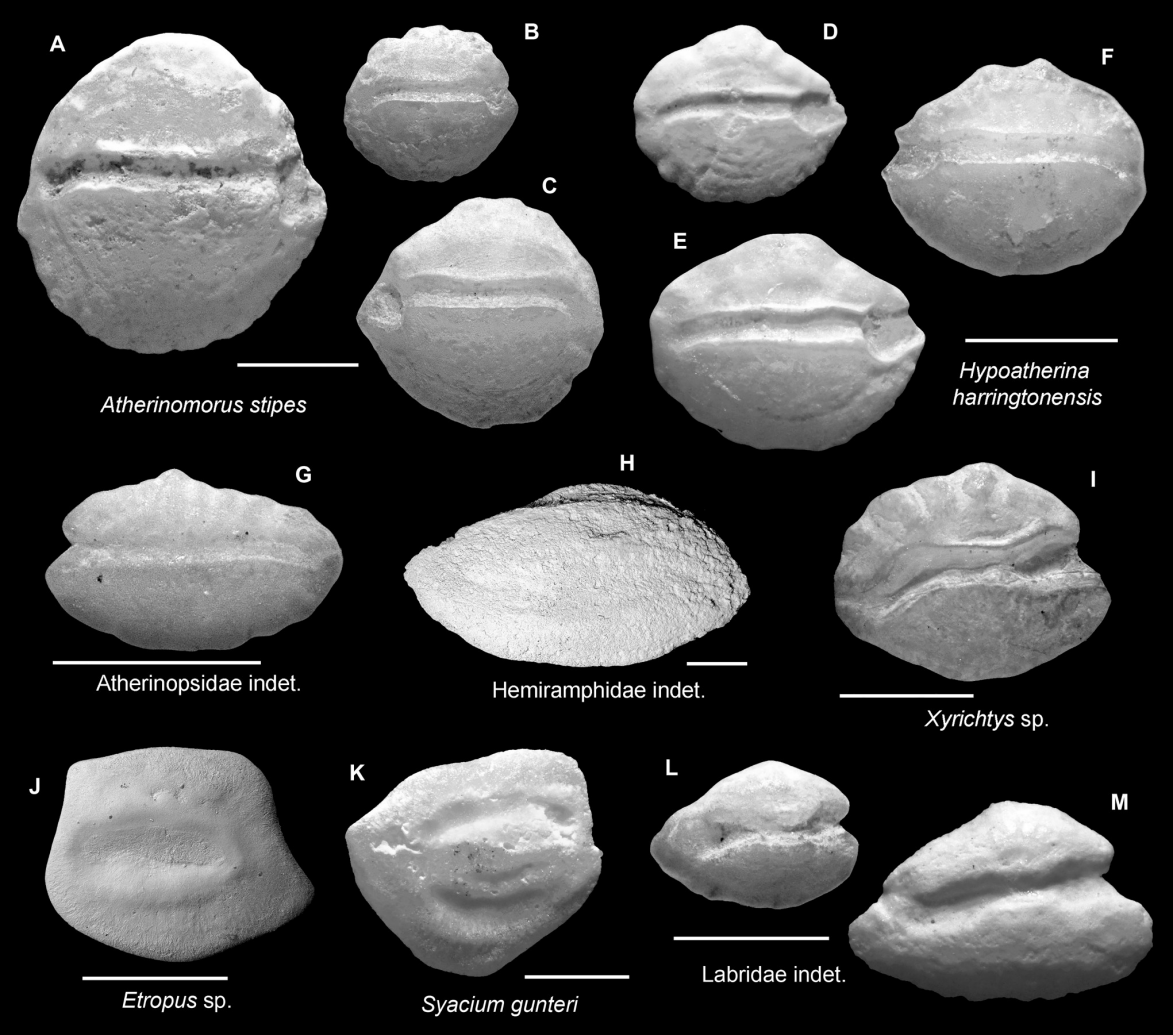
Fish otoliths from the mid-Holocene and sub-Recent reef sediments. Images are inner views and scale bars = 1 mm. **A-C.** *Atherinomorus stipes*, A, B, left otoliths, sub-Recent, Casa Blanca new; C, right otolith, mid-Holocene, Sweet Bocas Trench 7. **D-F.** *Hypoatherina harringtonensis*, sub-Recent, D, E, left otoliths, Airport Point; F, right otolith, Cayo Adriana. **G.** Atherinopsidae indet., right otolith, sub-Recent, Playa Estrella. **H.** Hemiramphidae indet., right otolith, sub-Recent, SW Bocas Island. **I.** *Xyrichtys* sp., left otolith, mid-Holocene, Duverge Road Section. **J.** *Etropus* sp., right otolith, sub-Recent, STRI Point. **K.** *Syacium gunteri*, left otolith, mid-Holocene, Las Clavellinas. **L, M.** Labridae indet., left otoliths, sub-Recent, L, Airport Point; M, Hickory Ship, La Caleta.

**Fig 7:**
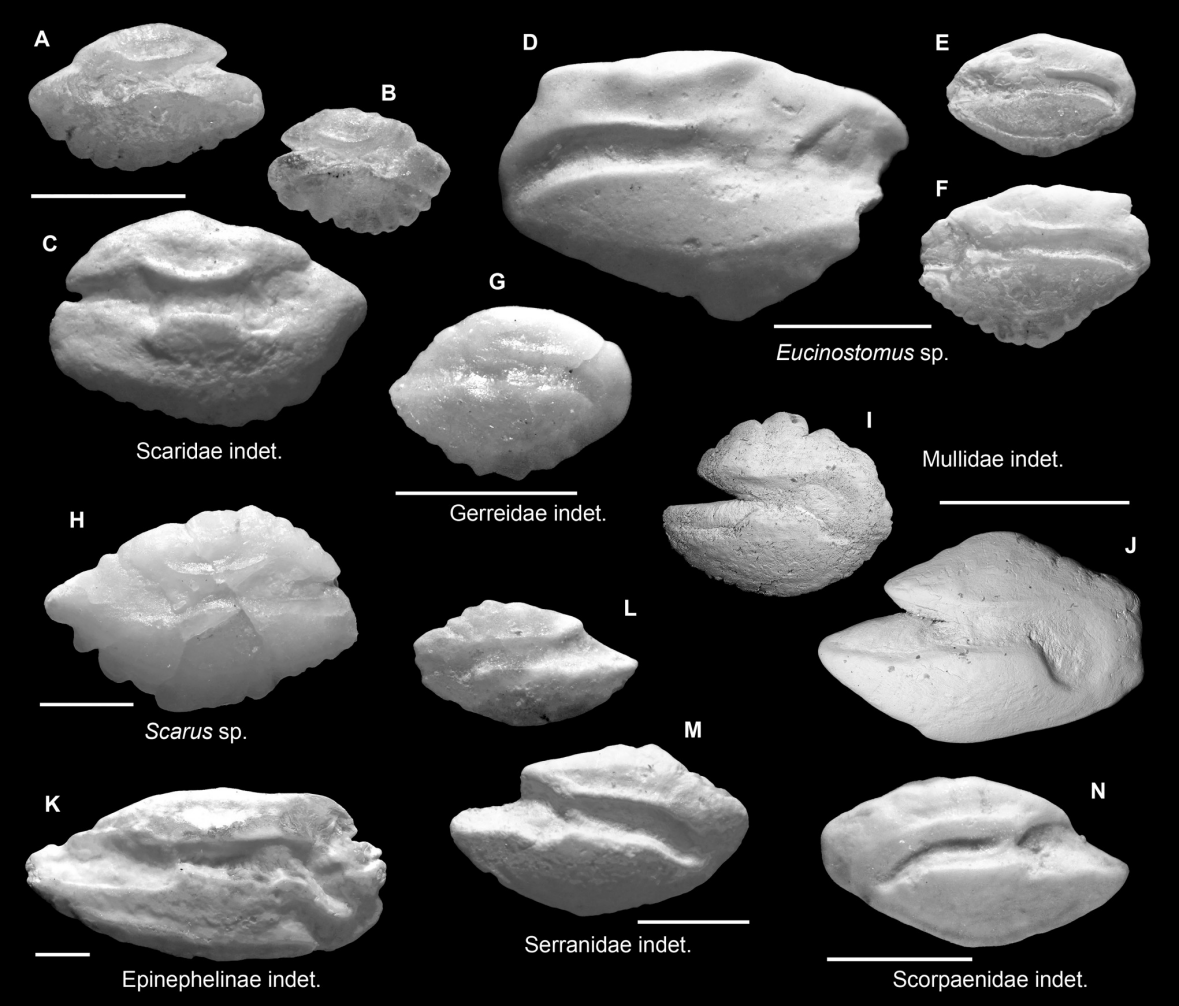
Fish otoliths from the mid-Holocene and sub-Recent reef sediments. Images are inner views and scale bars = 1 mm. **A-C.** Scaridae indet., A, left otolith, mid-Holocene, Sunset Point; B, right otolith, sub-Recent, Cayo Adriana; C, right otolith, sub-Recent, Punta Caracol. **D-F.** *Eucinostomus* sp., D, left otolith, sub-Recent, Cayo Adriana; E, F, right otoliths, mid-Holocene, E, Sweet Bocas Trench 3; F, Sunset Point. **G.** Gerreidae indet., right otolith, mid-Holocene, Sweet Bocas Trench 3. **H.** *Scarus* sp., left otolith, mid-Holocene, Sweet Bocas Trench 6. **I, J.** Mullidae indet., right otoliths, sub-Recent, I, La Caleta Beach Reef; J, Hickory Ship, La Caleta. **K.** Epinephelinae indet., right otolith, mid-Holocene, Cañon los Rios. **L, M.** Serranidae indet., sub-Recent, Cayo Adriana, L, left otolith; M, right otolith. **N.** Scorpaenidae indet., left otolith, sub-Recent, Cayo Adriana.

**Fig 8:**
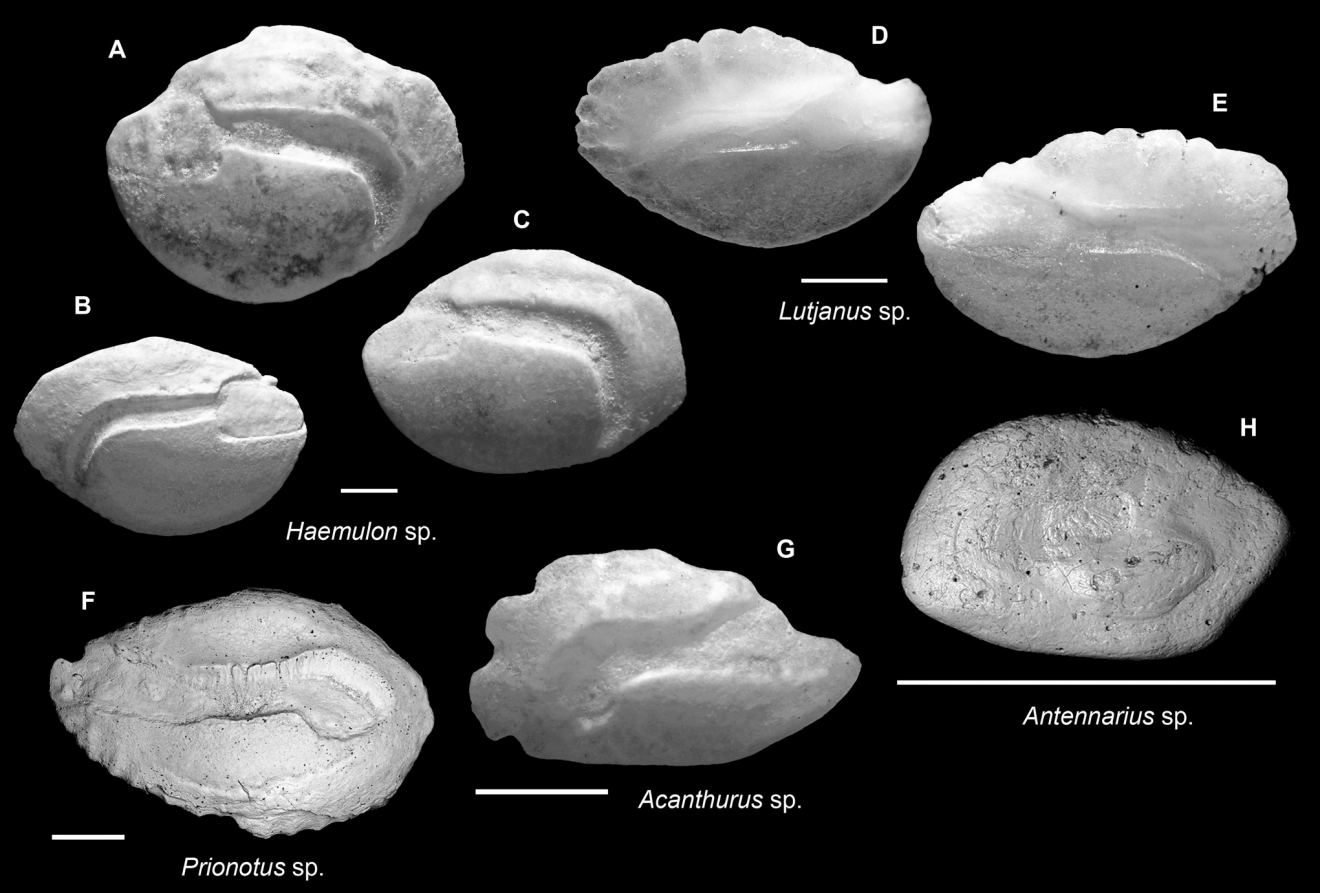
Fish otoliths from the mid-Holocene and sub-Recent reef sediments. Images are inner views and scale bars = 1 mm. **A-C.** *Haemulon* sp., A, C, right otoliths, mid-Holocene, Cañon los Rios; B, left otolith, sub-Recent, Feri, SW Levantado, Samana Bay. **D, E.** *Lutjanus* sp., D, left otolith, sub-Recent, SW Bocas Island; E, right otolith, mid-Holocene, Sunset Point. **F.** *Prionotus* sp., right otolith, mid-Holocene, Cañon de Buo. **G.** *Acanthurus* sp., left otolith, sub-Recent, Casa Blanca new. **H.** *Antennarius* sp., right otolith, sub-Recent, Wreck Limon La Caleta.

A list of otolith taxa recovered from fossil and sub-Recent sediment assemblages is presented in Table 3. Otoliths were identified to 35 families and 56 taxa (Figs 3–8). An effort for identifying juvenile and/or poorly preserved otoliths to a lower taxonomic level (genus and species) is usually avoided. The taxonomic remarks below are based on rare or important taxa wherever necessary. The following classification follows Nelson et al. [59], with the exception of Microdesmidae *sensu* Nelson [60] that we recognized here as a different taxon from Gobiidae based on otolith morphology (see below).

**Table 3:** Otolith-based fish taxa identified in this study, with number of identified specimens.

Family	Otolith taxa	Figure examples	Bocas del Toro		Dominican Republic		Number of otoltihs
			Holocene	sub-recent	Holocene	sub-recent	
Albulidae	*Albula vulpes*	Fig 3A	1				1
Congridae	*Ariosoma* sp.	Fig 3G				1	1
Engraulidae	Engraulidae indet.	Fig 3B and 3C	199	207	32	38	476
Clupeidae	*Harengula* sp.	Fig 3J	3	4			7
	*Jenkinsia* sp.	Fig 3D–3F	4	34			38
	*Sardinella* sp.	Fig 3H				1	1
	Clupeidae indet.	-	4	7		1	12
Synodontidae	*Saurida* sp.	Fig 3M	2	4		2	8
Myctophidae	*Diaphus* sp.	Fig 3K and 3L				7	7
	*Hygophum* sp.	Fig 3I				1	1
	Myctophidae indet.	-				2	2
Bregmacerotidae	*Bregmaceros* sp.	Fig 4A	7	13	39	2	61
Holocentridae	*Holocentrus* sp.	Fig 4B				1	1
	*Myripristis* sp.	Fig 4C				3	3
	*Ostichthys* sp.	Fig 4D				1	1
Carapidae	*Snyderidia canina*	Fig 4E		1		1	2
Ophidiidae	*Lepophidium* sp.	Fig 4F				6	6
	Ophidiidae indet.	-		1	1	18	20
Dinematichthyidae	*Ogilbia* sp.	Fig 4G	9	37	2	5	53
	*Ogilbichthys microphthalmus*	Fig 4J	3	8		9	20
Batrachoididae	*Amphichthys cryptocentrus*	Fig 4K and 4L	1	3			4
	*Porichthys plectrodon*	Fig 4H and 4I	1	1			2
	Batrachoididae indet.	-		4		1	5
Apogonidae	Apogonidae indet.	Fig 5A–5C	79	194	24	161	458
Gobiidae	Gobiidae indet.	Fig 5D and 5E	1183	1353	134	174	2844
Microdesmidae	Microdesmidae indet.	Fig 5F			1		1
Pomacentridae	Pomacentridae indet.	Fig 5G–5I	5	4		14	23
Opistognathidae	*Lonchopisthus* sp.	Fig 5J–5L	3	5			8
	*Opistognathus* sp.	Fig 5N		1		1	2
Blenniidae	Blenniidae indet.	Fig 5M		1	1		2
Labrisomidae	Labrisomidae indet.	Fig 5O and 5P	1	2		8	11
Atherinopsidae	Atherinopsidae indet.	Fig 6G	1	2			3
Atherinidae	*Atherinomorus stipes*	Fig 6A–6C	2	46		3	51
	*Hypoatherina harringtonensis*	Fig 6D–6F	4	133		6	143
	Atherinidae indet.	-	1	15		1	17
Hemiramphidae	Hemiramphidae indet.	Fig 6H		1			1
Paralichthyidae	*Etropus* sp.	Fig 6J		1			1
	*Syacium gunteri*	Fig 6K			1		1
Bothidae	Bothidae indet.	-	1		1		2
Labridae	*Xyrichtys* sp.	Fig 6I			1		1
	Labridae indet.	Fig 6L and 6M	5	10	1	6	22
Scaridae	*Scarus* sp.	Fig 7H	2				2
	Scaridae indet.	Fig 7A–7C	5	11		2	18
Gerreidae	*Eucinostomus* sp.	Fig 7D–7F	14	9			23
	Gerreidae indet.	Fig 7G	3	3			6
Mullidae	Mullidae indet.	Fig 7I and 7J				10	10
Serranidae	Epinephelinae indet.	Fig 7K			1		1
	Serranidae indet.	Fig 7L and 7M	1	5	2	4	12
Haemulidae	*Haemulon* sp.	Fig 8A–8C		10	6	3	19
	Haemulidae indet.	-	3	9		4	16
Lutjanidae	*Lutjanus* sp.	Fig 8D and 8E	1	1			2
	Lutjanidae indet.	-				1	1
Scorpaenidae	Scorpaenidae indet.	Fig 7N		1			1
Triglidae	*Prionotus* sp.	Fig 8F			1		1
Acanthuridae	*Acanthurus* sp.	Fig 8G	1	1			2
Antennariidae	*Antennarius* sp.	Fig 8H				1	1
			1549	2142	248	499	4438

### Engraulidae (Fig 3B and 3C)

Our engraulid otoliths show variations in the posterior rim, otolith length-height ratio, and the length of rostrum suggesting that more than one species or genus are involved. Indeed, ca 13 species occur in the Bocas region (western Panama) [61]. Reference specimens of this group are far from sufficient for a reliable taxonomic conclusion and otoliths of closely related genera and species exhibit similar features precluding clear identification, e.g., *Anchoa* and *Anchovia* [36]. We, therefore, maintain identification at the family level only.

### *Jenkinsia* (Clupeidae) (Fig 3D–3F)

The otoliths of *Jenkinsia* are trapezoid in shape, with a deep and triangular ostium and a shallow, indefinite cauda. They have a robust rostrum but lack a pronounced antirostrum, resembling much to those of *Etrumeus*. The combination of these features make them readily distinguishable among clupeid otoliths, but very difficult to discriminate at the species level. To our knowledge, images of *Jenkinsia* otoliths have never before been published.

### *Porichthys plectrodon* (Batrachoididae) (Fig 4H and 4I)

The otoliths of *Porichthys* share many superficial similarities with those of gobiids; their otoliths are high-bodied with a centrally-located sulcus and a collicular crest on the crista inferior of the cauda. The diagnostic features for *Porichthys* otoliths compared to those of Gobiidae are a much wider and longer sulcus, a marked constriction at the collum of crista superior, and a wider ventral rim relative to the dorsal rim. The varied shapes in the dorsal rim of our *Porichthys* specimens, each from the mid-Holocene and sub-Recent sample of Bocas, is here considered ontogenetic differences [62]. They are very similar to the upper Miocene (Cercado Formation) *Porichthys* otoliths from the Dominican Republic [62]. We identified our specimens as *Porichthys plectrodon* based on recent comparative material (W. Schwarzhans, unpublished data).

### Gobiidae and Microdesmidae (Fig 5D–5F)

Although otoliths of this taxon are the most abundant in our samples and they are readily distinguished at the family level, the lack of available comparative material does not allow a confident identification to genus or higher at the current time. Therefore, we presented all gobiid otoliths at family level in Table 3. The two figured gobiid otoliths are well-preserved and they represent the otoliths of *Bathygobius* (Fig 5D) and *Bollmannia* (Fig 5E), based on recent comparative material (W. Schwarzhans, unpublished data). Apart from apparent interspecific differences in their morphology, the rotenone sampling survey also reveals that intraspecific variation, including ontogenetic change, can be great (e.g., S5 and S6 Figs), which adds further difficulties to correct identification.

Two genera of Microdesmidae, *Microdesmus* and *Cerdale*, have been reported in the greater Caribbean [46] but because sufficient reference otolith specimens are currently not available, we assigned our specimens to the family rank. The otoliths of Microdesmidae are also characterized by a typical gobiid sulcus, however, their outline shape, which is a bowling-shaped, tall body (Fig 5F) [23], differs drastically from all other Gobiidae that is usually rectangular or square in shape. Here, for the purpose of demonstrating the greatest diversity of otolith collections, we regard Microdesmidae as a separate taxon from other gobiids based on otolith. This classification scheme is identical as in Nelson [60], though recently Nelson et al. [59] recognized Microdesmidae as being members of the family Gobiidae.

### Pomacentridae (Fig 5G–5I)

Ontogenetic variation in otolith shape within the family Pomacentridae is high. Otoliths of juveniles are typically short and tall while those of adults are more elongate and usually possess a pointed spine in the middle of the dorsal rim. However, the otoliths are conservatively characterised by a triangular ostium that is bordered by an oblique and strongly developed crista superior and a straight crista inferior (e.g., S4 Fig). The majority of the otoliths observed in our sediment assemblages belong to juvenile fish, although some large and well-preserved specimens likely belong to the genus *Stegastes* (Fig 5I) which is common on Caribbean coral reefs. Nonetheless, we prefer to retain our identification to the family level until the reference collection is more complete.

### Blenniidae and Labrisomidae (Fig 5M, 5O and 5P)

Correct identification of otoliths in these groups can be problematic because of their small size. Otoliths are more or less triangular and can be confused with poorly-preserved otoliths from Labridae and Tripterygiidae. In labrisomid otoliths, the sulcus is deep, there is an ostial colliculum but without an evident elevation at the collum, and the posterior margin of the cauda is usually indeterminate (S5 Fig). On the other hand, the otoliths of Blenniidae are, as seen in those of Labridae, more compact in shape and having a shorter and more elevated collum; their cauda, with regard to that of labrids, is often very wide with its tip markedly bent (S5 Fig).

### Atherinopsidae (Fig 6G)

Our atherinopsid otoliths are very elongate and possess a straight sulcus with a cauda that is bent very slightly at the end. They resemble some of the mugilid otoliths [23], but their pointed dorsal rim is diagnostic to the family Atherinopsidae whereas in the mugilid otoliths the dorsal rim is typically higher at the anterior portion.

### *Xyrichtys* (Labridae) (Fig 6I)

The remarkable *Xyrichtys* otoliths are hexagonal in shape and have a slightly elevated collum. While their shape makes them distinct from the otoliths of other wrasses [52,63], the elevated collum is a typical feature for labrid otolith. The curvature of the cauda is variable, and likely related to ontogeny [39].

### *Antennarius* (Antennariidae) (Fig 8H)

The otoliths of *Antennarius* are highly variable in terms of their outline shape but are often blunt and much thicker at the posterior end than at the anterior. The most distinct features of the *Antennarius* otoliths are perhaps the swelling ventral area and an enclosed, centrally-located sulcus, which can be observed in this specimen.

## Results

### Otolith abundance and preservation

**Fig 9:**
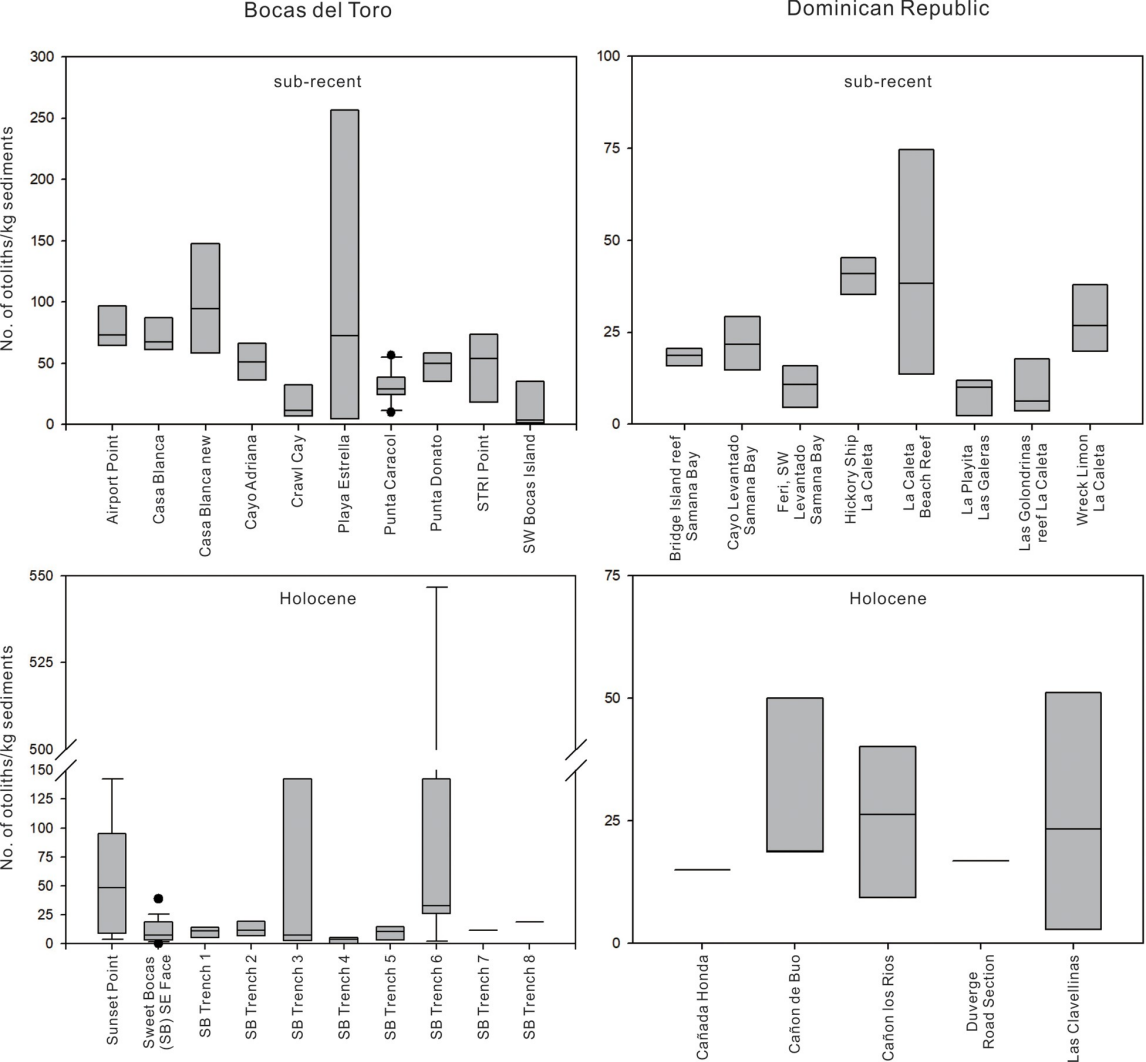
**Differences in the densities of otoliths per kg of reef sediment in sites from sub-Recent (top) and mid-Holocene (bottom) coral reefs in Bocas del Toro, Panama (left) and the Dominican Republic (right).** Box plot with lower (25th percentile), median and upper (75th percentile) boundaries, whiskers of 10th and 90th percentiles, and outliers (solid circle) outside of 10th and 90th percentiles are presented.

Using the reference collection and other guides (see Materials and Methods), we identified a total of 5,413 otoliths that we isolated from the 500 μm fraction of modern and mid-Holocene reef sediments from Bocas del Toro and the Dominican Republic (Table 1). The average density of otoliths across all samples was 44.53 kg^-1^ of reef sediment (SD = 73.15 kg^-1^). Average density of otoliths was marginally higher (Mann-Whitney U = 23, p = 0.0536) in sub-Recent than mid-Holocene sediments in Bocas del Toro (56.00 kg^-1^ and 48.57 kg^-1^, respectively, S3 Table), but average densities between for both ages was similar (Mann-Whitney U = 6, p = 0.8259) in Dominican Republic samples (22.82 kg^-1^ and 24.18 kg^-1^, respectively, S3 Table). When sub-Recent and mid-Holocene data were pooled, densities of otoliths were similar (Mann-Whitney U = 76, p = 0.1556) in Bocas del Toro (Mdn = 39.6) and the Dominican Republic (Mdn = 21.9). Sub-Recent data, otolith densities in the Dominican Republic (Mdn = 20.1) were found to be different than those from Bocas del Toro (Mdn = 50.3) (Mann-Whitney U = 12, p = 0.0147). Variation across all samples was in general very high (Fig 9, S3 Table). For example, three samples from the Holocene Sweet Bocas Trench 6 reef contained more than 390 otoliths kg^-1^ of sediment but one sample from the same site contained less than five otoliths. This could be driven by the relative proportion of coral and other skeletal remains, and demonstrates the need for many replicate samples. Otolith densities at Playa Estrella (sub-Recent Bocas del Toro) differed substantially from other sites (Fig 9), presumably because they were collected from soft sediments with corals rather than fringing reef environments. Preservation of otoliths was, on the whole, very good with over 75% of otoliths being assigned a taxon (Table 1, S7 Fig). The majority of otoliths that could not be identified belonged to juveniles, or less frequently, broken and/or poorly-preserved.

### Richness and diversity

**Fig 10:**
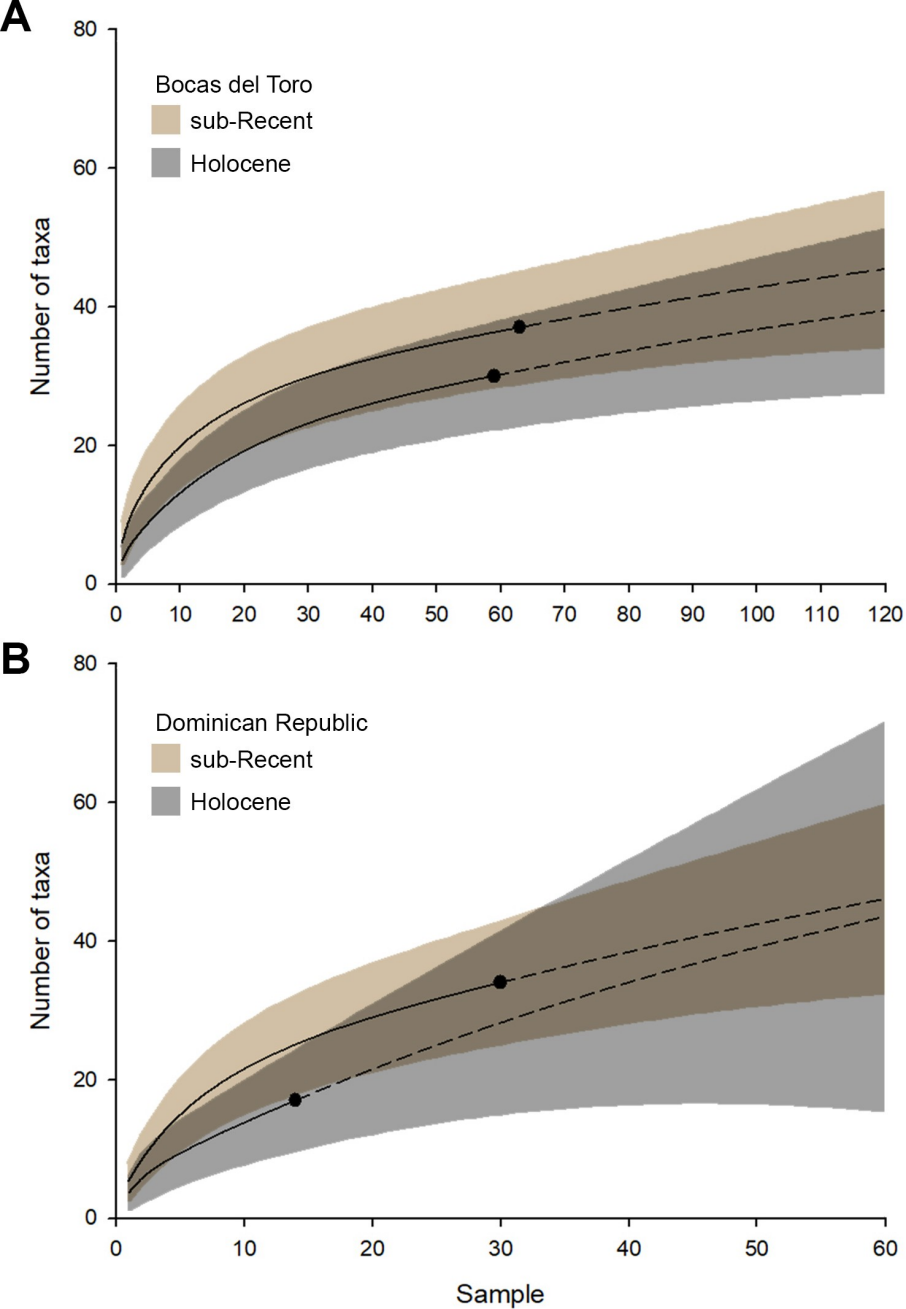
Sample-based rarefaction and extrapolation curves with 95% confidence intervals. Reference samples are indicated by solid circles, rarefaction by solid lines, and extrapolation by dashed lines.

Rarefaction curves calculated from both sub-Recent and mid-Holocene samples from Bocas del Toro reached close to asymptotes. Extrapolation estimated that the number of taxa would not increase substantially with greater sampling (Fig 10A). Similar patterns were observed in the less-well sampled sub-Recent material from the Dominican Republic, but a more intensive sampling would be required for the mid-Holocene Dominican Republic material to confidently estimate the total number of taxa (Fig 10B).

Both rarefaction and Shannon’s and Simpson’s estimators (S4 Table) suggested that, in both regions, sub-Recent samples have greater diversity compared to mid-Holocene samples. Pielou's evenness index suggested that mid-Holocene Bocas del Toro assemblages were strongly dominated by few taxa compared to their more evenly represented sub-Recent counterparts, whereas evenness was similar in mid-Holocene and sub-Recent Dominican Republic samples (S4 Table).

### Rank-abundance of taxa

**Fig 11:**
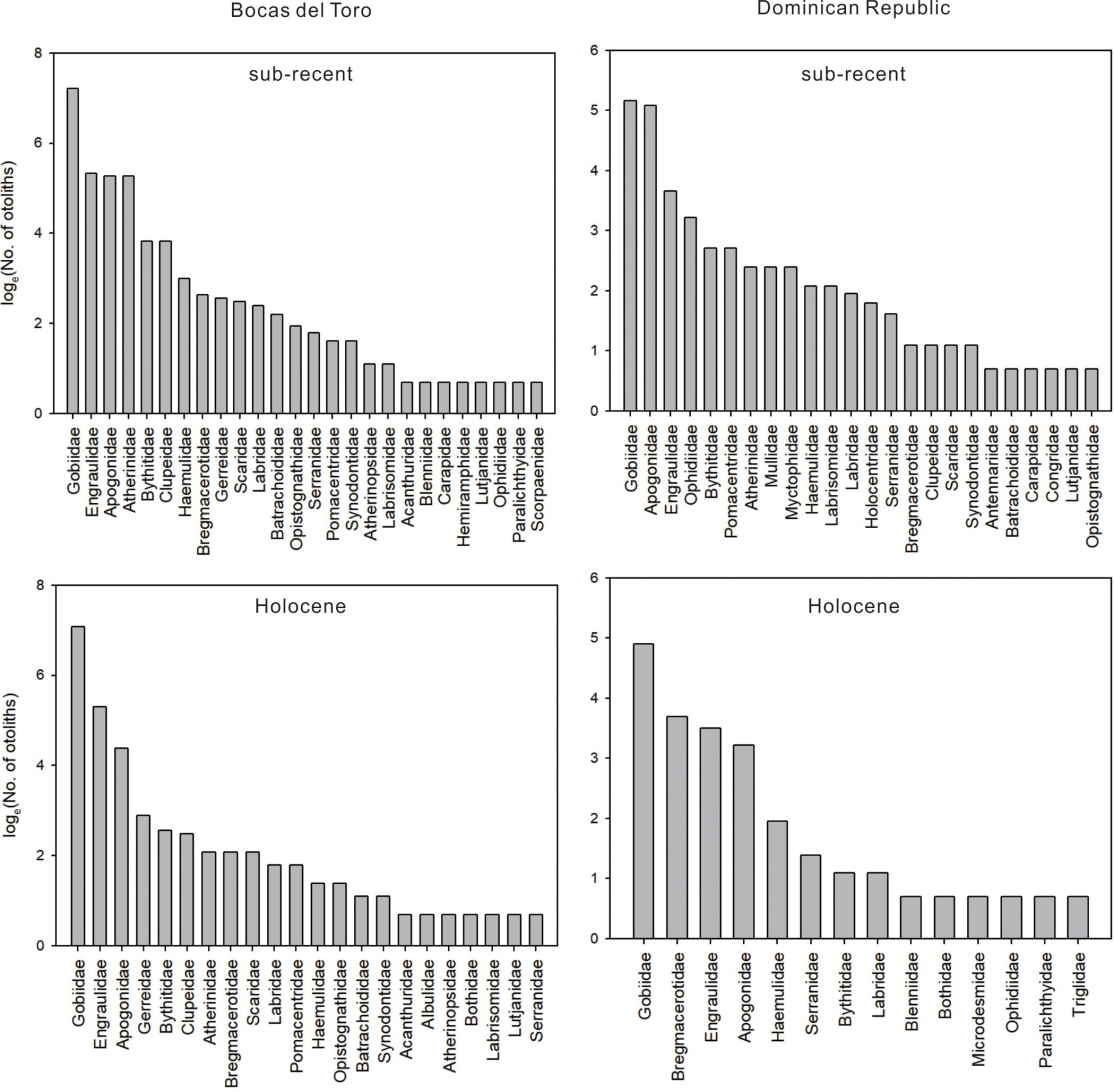
**Rank-abundance of otolith families in sites from sub-Recent (top) and mid-Holocene (bottom) coral reefs in Bocas del Toro, Panama (left) and the Dominican Republic (right).** Note that otolith counts are log-transformed.

The composition and abundances of otolith families demonstrate that fishes of small size were a dominant element in all assemblages. Larger fishes were always rare (Fig 11, Table 3). The gobies (Gobiidae) were the most numerically abundant family in 26 out of the total 33 sites. Anchovies (Engraulidae) were the dominant family in two sites in Holocene Bocas del Toro and one site in sub-Recent Dominican Republic, and cardinalfishes (Apogonidae) dominated one site in sub-Recent Bocas del Toro and three sites in sub-Recent Dominican Republic.

Mid-Holocene and sub-Recent rank-abundances were more similar in Bocas del Toro than in the Dominican Republic (Fig 11). The top three same families (Gobiidae, Engraulidae, and Apogonidae) comprised over 80% of both sub-Recent and mid-Holocene assemblages in Bocas del Toro, whereas rank order and proportion differences were evident between ages in the Dominican Republic assemblages (Fig 11). Codlet otoliths (Bregmacerotidae) were the second most abundant family in the mid-Holocene Dominican Republic, while they were less abundant in the sub-Recent Dominican Republic samples (Fig 11).

### Rotenone surveys

A total of 388 fishes (12 families and 26 taxa) were captured on four reefs (Fig 1A) in Bocas del Toro (Table 2, S5 Table) using rotenone station surveys (see Materials and Methods) and compared to otolith sediment assemblages from the same sites. We compared at a taxonomic level applicable to the otolith assemblages and excluded pelagic fishes that are missed by the rotenone surveys. The majority of fishes captured in rotenone stations were small cryptobenthic fishes, including members of gobies (Gobiidae) and cardinalfishes (Apogonidae). Anchovies (Engraulidae), herrings (Clupeidae) and other pelagic fishes were not captured in rotenone surveys.

**Fig 12:**
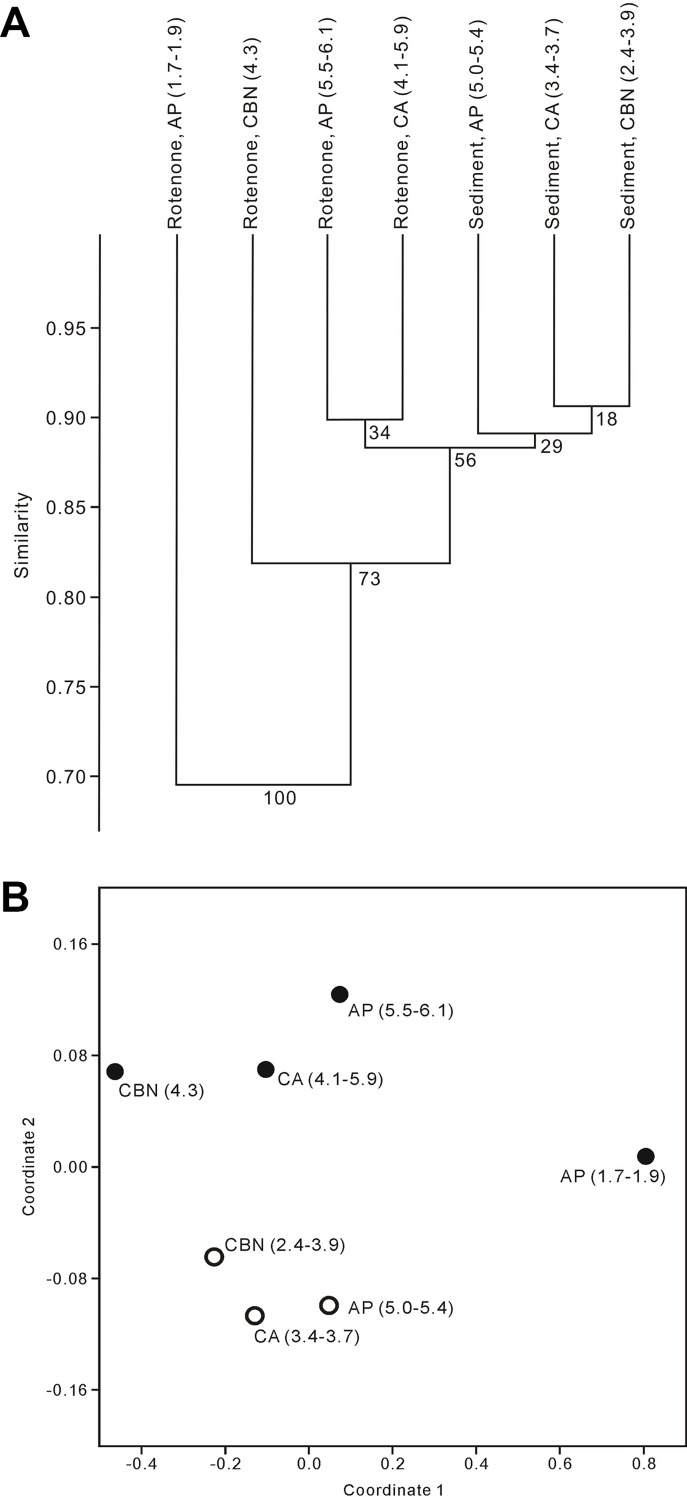
Comparison of rotenone samplings and sub-Recent sea bottom otolith assemblages on four reefs in Bocas del Toro. A cluster analysis **(A)** and a non-metric multidimensional scaling **(B)** with Bray-Curtis similarity index were performed. Bootstrap values are given at the roots of each cluster. Samples less than 10 fish individuals are excluded. Depths are indicated in the parenthesis. Empty circle, sediment assemblages. Solid circle, rotenone samplings. AP, Airport Point. CA, Cayo Adriana. CBN, Casa Blanca new.

Both cluster and nMDS analyses revealed that the composition of the standing living community at the shallowest reefs was distinct from the deeper reefs (Fig 12). In cluster analysis, Bray-Curtis similarity between rotenone surveys and sediment otolith assemblages was high for all samples compared (> 0.8), except for two shallowest (1.7–1.9 m) rotenone samples forming a distinct cluster (Fig 12A). Likewise in nMDS, the first coordinate clearly separated shallow from the deep samples (Fig 12B). The second coordinate in nMDS, on the contrary, likely distinguished rotenone samples from sediment otolith assemblages (Fig 12B). The stress value (0.04) of our nMDS implies, however, very little loss of information.

### Estimating time-averaging in modern otolith assemblages

**Fig 13:**
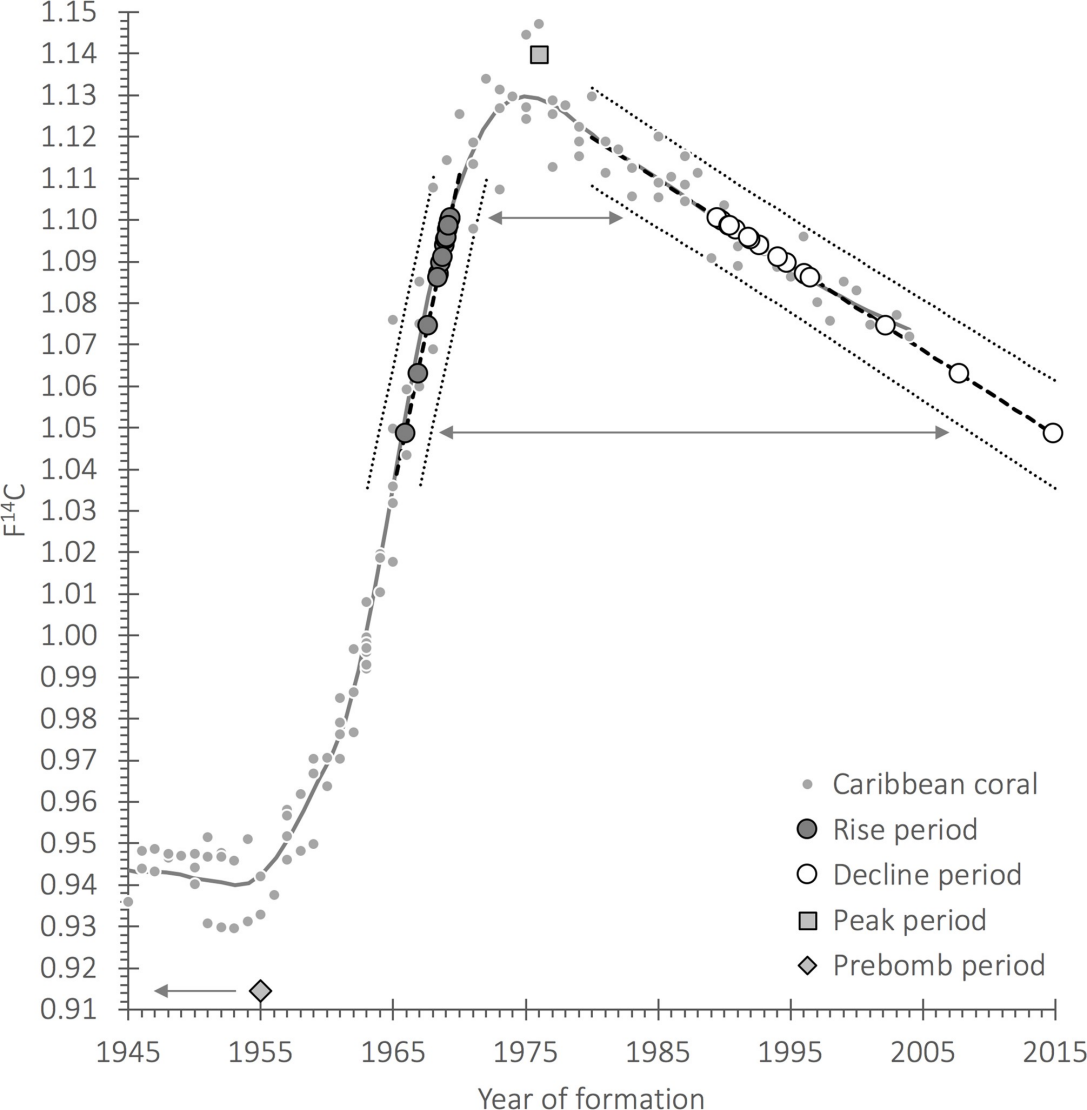
Plot of the radiocarbon (^14^C) reference records available from hermatypic corals in the Caribbean Sea (Loess curve fit) with otolith ^14^C data from sediment cores plotted on the correlated dates of formation. For most measurements the values crossed the regional ^14^C reference curve with potential dates from the bomb ^14^C rise or decline periods, with the exception of two samples (S1 Table). These fish were a short-lived species (gobies, Gobiidae) and posited to cover a negligible age (<2 years of growth). Dates of formation for rise and decline periods were from a simple linear regression of coral ^14^C values that were coincident with the Loess curve for each time span. The outliers were one ^14^C value that is centered on the peak at 1976 and the other is a pre-bomb otolith that was aged to median year of formation of 1550 AD (2 sigma = 1490–1640; CALIB Rev7.1.0 and see S2 Text).

We used radiocarbon dating directly on 20 otoliths to explore the amount of time encapsulated by a single bulk sample sediment otolith assemblage from a modern, actively accreting, reef. Nineteen of the otoliths were of a modern origin (> 1950) (S1 Table). We found that ^14^C values were well above mean pre-bomb levels (mean F^14^C = 0.942 ± 0.015) covering an F^14^C range of 1.05–1.14 and could be assigned a calibrated date from either the rise or decline periods (Fig 13). Dates from the rise period were 1965.9–1969.3 and from the decline period were 1989.6–2014.8 using linear regressions that were coincident with the Loess central tendency (rise: Year = 0.01521*F^14^C – 28.86, R^2^ = 0.904 and decline: Year = –0.002046 + 5.171, R^2^ = 0.858). One value was near the peak of the coral records and could be assigned a median year of ~1976 and may have had an uncertainty of ± 6 years at most from the observed range of peak values (~1970–1982). One pre-bomb value lying below mean pre-bomb levels (F^14^C = 0.9144 ± 0.0024) and aged using ^14^C dating, revealed a median date of 1550 AD after correction for regional reservoir age. Potential dates of formation were 1490–1640 AD (2 sigmas). Thus, 19 out of 20 otoliths could be assigned ages of between ~1966 and 2014, while one otolith was dated to 1550 AD.

## Discussion

### Abundance and taxonomic resolution

Our findings reveal that otoliths can be abundant and well-preserved in unconsolidated carbonate-rich reef sediments. We propose that these records can be used to extend monitoring of reef fish communities to complement ongoing research into the spatial and temporal variation in the biodiversity and ecological functioning of coral reefs. This work augments ongoing research on other skeletal elements preserved in reef matrices including molluscan shells [28], sponge spicules [64,65], fish teeth [6], urchin spines [66], and shark dermal denticles [67] to expand our understanding of variation and change in whole reef communities.

High-level taxonomic identification of otoliths often relies on features that appear only in adult individuals [24]. The high proportion of juvenile otoliths in our assemblages therefore required us to be very conservative in our identifications (see Systematics). While we were able to identify 82% of otoliths to the family level, only about 10% of them were assigned to a genus or a species. Eighteen percent of otoliths could not be confidently identified to any taxon, typically because they were prohibitively small. For example, otoliths from juvenile porgies (Sparidae) and snappers (Lutjanidae) are so similar [51] to prevent confident distinction, as are juveniles within the family Gobiidae (S5 and S6 Figs) and Apogonidae (S3 Fig), identification below family rank is practically not possible without adult otoliths available. The problem of limited diagnostic information in juvenile fish otoliths is not restricted to those retrieved from reef sediments [24], although the high proportions of juveniles we recovered, compared to open-water sediments, makes it particularly challenging. While some fishes do inhabit reefs as juveniles and migrate to a more pelagic setting as they age [68], the dominance of juvenile otoliths was a pattern observed across all families [69]. This pattern is, therefore, more likely caused by high rates of juvenile mortality through the predation of small fishes, which is documented to be intense on reefs [69]. As we discuss later, we suspect that a large proportion of the otoliths in reef sediment assemblages arrive there via ingestion and defecation. Ongoing work to improve the reference collection, with a greater focus on early ontogenetic sequences of underrepresented taxa, will likely improve our ability to confidently identify juvenile otoliths.

### Collection power, taxon richness and biodiversity

Rarefaction curves and extrapolation suggest that sampling effort was, on the whole, fairly good, although mid-Holocene sites from the Dominican Republic are currently under-sampled (Fig 10). Given this caveat if were to take the results at face value, our data suggest that richness and diversity were lower in the mid-Holocene than the sub-Recent reefs in both Bocas del Toro and the Dominican Republic (Fig 10, S4 Table). This intriguing preliminary pattern could be explained by taphonomic processes, ecological shifts and/or anthropogenic impacts, amongst others. Further studies are required to better understand the processes of otolith assemblage accumulation and burial on coral reefs. However, given that a similar change in diversity over time is observed in both Panama and the Dominican Republic, a common driver may be responsible. Though it is likely reflecting changes in the cryptobenthic fish fauna driven by a decline in structural complexity of coral reefs due to pervasive historical and contemporary human disturbances, we continue research to explore possible explanations for why modern reefs appear to harbour more diversity than their mid-Holocene counterparts.

### Taxonomic, functional and ecological representation

Otoliths representing a wide spectrum of reef fishes were recovered from our sediment otolith assemblages, including 35 identified families, of which we were able to resolve 56 taxa (Figs 3–8, Table 3). The major taxonomic and functional components of a characteristic Tropical West Atlantic reef fish community are represented [70] with some notable exceptions. Chondrichthyans, for example, have no otolith, although dermal denticles preserve well in reef sediments [67]. Groups with otoliths < 500 μm in size (e.g., Chaenopsidae) were absent as expected given that our samples were sieved at 500 μm. In addition, otoliths of Tetraodontidae are very fragile and were also absent.

The otolith assemblages were dominated by small fishes, with gobies (Gobiidae), anchovies (Engraulidae), and cardinalfishes (Apogonidae) always in the top taxa (Fig 11). Larger, typical reef fishes (e.g., parrotfishes, angelfishes, and butterflyfishes) were up to three orders of magnitude less abundant (Fig 11, Table 3). The representation of otolith assemblages here is not an artifact of size of the otoliths—that larger otoliths are potentially more prone to breaking and hence lost in the sediments—because otolith size is more related to the phylogenetic history of the fish it belongs to than to the size of the fish it originated, except in an ontogenetic sense. Small cardinalfishes, for example, have larger otoliths than those of wrasses (Labridae) even though the wrasse may have a larger body size.

The dominance of small cryptobenthic and epipelagic fishes in our sediment assemblages is in sharp contrast to the findings of visual surveys that are frequently dominated by large diurnal fishes. For example, Dominici Arosemena and Wolff [71] and Seemann et al. [72] used visual surveys to quantify reef fishes in Bocas del Toro, yet failed to record any cryptobenthic cardinalfishes or epipelagic anchovies. Abundance data from visual surveys and otolith assemblages are therefore not directly comparable. We propose that the sediment otolith assemblages capture a more representative time-averaged survey of small fishes absent in traditional visual surveys. This conclusion is supported by the high abundance of cryptobenthic fishes in our rotenone surveys (S5 Table) and the fact that small epipelagic fishes (e.g., anchovies) were routinely observed swimming around the reefs. Ackerman and Bellwood [73] and more recently Brandl et al. [69] demonstrated that clove oil and rotenone in enclosed treatments better captures the standing reef fish community than visual surveys. These studies reveal that cryptobenthic fishes often match or dominate reefs in terms of number of fish species and abundance of individuals. Because their turnover and growth rates are considerably higher, cryptobenthic fauna can contribute significantly to *per capita* energy flow in reefs [69,74] explaining why these small fishes dominate the otolith sediment assemblages. Similar observations have been made in otolith assemblages from deep-sea soft sediments, where numerically abundant short-lived, small mesopelagic taxa (e.g., Myctophidae) dominate rare and large benthic-benthopelagic fish that are at a higher trophic level (e.g., Macrouridae) [25–27]. Thus, the time-averaged accumulation of otoliths in sediments and their identification to ecological guilds could be a powerful approach for exploring controversial questions about the trophic structure of coral reefs [75].

Our comparison between surveys in rotenone stations and the otolith assemblages in sediments from the same sites, reveals a high concordance between the two approaches (Fig 12). Both reveal comparably high-dominance of the cryptobenthic fauna, in terms of abundances. Coral reef fish communities are characterised by very high taxonomic diversity but with few dominant taxa and many rare taxa [42]. The otolith assemblages document such uneven relative abundance, further supported by our small rotenone samplings and other similar studies in the area [76]. The shallowest two rotenone samples that different from other samples in terms of taxonomic composition and relative abundance (Fig 12) may involve factors other than depth only, but our limited sample size does not allow a conclusive explanation, further studies focusing on this topic are necessary. Though preliminary in scope, our rotenone samplings permit a unique opportunity to explore the link between fish biocoenosis and otolith thanatocoenosis for the first time.

Anchovies, herrings, and silversides are appropriately represented in the otolith sediment assemblages but are unsurprisingly absent in the rotenone surveys because these fast epipelagic fish swim around living reefs [46,77], avoid divers and are not expected to be caught in the nets of a rotenone survey [44]. We conclude that otolith assemblages in reef sediments are an ecologically representative accumulation of entire water column fishes condensed into a single layer in the sediments and time averaged.

### Preservation and taphonomic processes

The otolith assemblages recovered from sub-Recent and mid-Holocene Dominican Republic and Caribbean Panama bulk-sampled reef sediments were, on the whole, well-preserved. The proportion of eroded and unidentifiable specimens was fairly consistent across samples and sites (S7 Fig). When otoliths could not be identified it was usually because they were juvenile rather than poorly-preserved. In all samples, irrespective of age, eroded and pristine-looking otoliths were found together in apparently similar proportions, suggesting that erosion occurs pre-burial, such as through digestion by predators. If erosion were post-burial all otoliths might be expected to be affected equally unless time averaging was high (see below). Otoliths of both mid-Holocene and modern samples were not stained orange or brown, as occurs in older material, suggesting a minimal chemical alteration. This is supported by x-ray diffraction studies of coral fossils from the same mid-Holocene sites revealing almost entirely diagenetically-unaltered preservation [28,78]. Otoliths in both sub-Recent and mid-Holocene samples appear to have undergone rapid burial because no encrusting epibiota was observed, contrasting with the frequently-encrusted otoliths found in deep-sea sediments where burial is slower [27,79].

All otolith assemblages recovered from seafloor sediments represent time-averaged accumulations. The amount of time-averaging depends on sediment accumulation rate, reef accretion rate, the depth of digging into the framework when sampling, and amount of mixing from bioturbation or wave or current activity [80]. U-Th dating on dead coral pieces found in the top 5 cm on actively-accreting reefs in Bocas del Toro show corals can be as old as 1926 [6] but the majority date within the last 20 years. We found a similar pattern using bomb ^14^C dating on the otoliths themselves. Nineteen of the 20 otoliths we analysed are estimated to have dated from between ~1966 to 2014 (Fig 13), suggesting that the interlocking framework of small branching coral that restricts vertical movement of corals [6] also protects fine-grained reef matrix sediments between them where the otoliths accumulate. Bioturbation by burying shrimp [81], holothurians [82], and batoids [83] is frequently observed in loose sediments away from the reef framework, and this has been demonstrated to cause high time averaging of reef sediments [80], but these processes have not been observed in the reef proper where the coral framework deters bioturbators. Nevertheless, we did find one otolith that was potentially as old as 450 years (S1 Table). We, therefore, conclude that the majority of otoliths accumulating in actively accreting branching coral reefs are modern, although a small component of the otolith assemblage may be much older.

### How do otolith assemblages accumulate in reef sediments?

There are two main routes by which otoliths may become incorporated into reef sediments. First, *in situ* (on the reef) mortality of a fish, from disease or old-age, and the subsequent decay of its skeleton would lead to the release of the otoliths and their inclusion into the reef matrix. This process may be important in fishes or ecosystems that experience low rates of predation. However, predation on reef fishes is well documented to be extremely intense [84], especially on small-bodied fishes [69] where predation-driven mortality rates of over 7% per day have been observed [85].

Intense predation on reef fishes is supported by the fact that the majority of otoliths we observe in reef sediments are from juveniles. It is therefore likely that the overwhelming mode of incorporation of otoliths into reef sediments is through digestion followed either by regurgitation or defecation by predators. Three main groups of predators could be responsible; predatory fishes (including sharks), marine mammals, and seabirds. Epipelagic fishes, such as anchovies and silversides, likely experience intense predation from all three [86], whereas cryptobenthic fishes are major components of the diets of predatory reef fishes [69], and the stomach contents of reef-associated dolphins [87] and sharks show that they frequently prey upon reef fishes [88,89].

Predation by highly mobile predatory fishes, marine mammals and seabirds could, therefore, bring otoliths from other habitats onto reefs where they could become buried in the sediments. Dolphins [90] and reef-associated sharks [91,92] move between pelagic and reef systems subsidising reefs with nutrients from pelagic zones [93] and therefore potentially transferring digested otoliths from open water into reef sediments. This process could be responsible for the small proportion of exclusively open water pelagic taxa such as codlets (1.1%) and lanternfish (0.2%) we observe in the reef sediment otolith assemblages (Fig 11, Table 3). Indeed, fishes of both families are commonly found in the stomach contents of marine mammals like dolphins [94]. The occurrence of these open water pelagic taxa in reef sediments therefore likely reflects the strength of activity of sharks and marine mammals between open ocean and reef habitats rather than their occurrence on the reef itself. Seabirds likely play a less important role in the movement of otoliths into reefs than predatory fishes and mammals on Caribbean reefs because they are less abundant and often regurgitate pellets containing otoliths or defecate at roosting sites [95] rather than above reefs. Nonetheless, without a comprehensive understanding of the movement patterns of all potential predators and their food items from stomach contents, it is difficult to quantify the role predation and movement on the inclusion of external otoliths into reef sediments. We do however predict that the process is likely to vary considerably depending on the abundances of predators, the life history and life mode of prey items and the local topography and oceanographic settings of the coast [96]. We recommend further future work that should include a compilation of published research on movements of predators and their diets from stomach contents and other techniques, to provide greater insights into the role predation plays in the accumulation of otoliths into sediments. For example, to investigate the degree to which trophic physiological relationships changed from the mid-Holocene to today using carbon and nitrogen isotopes from otoliths [97–99].

## Conclusions

We describe a novel approach to reconstruct coral reef fish assemblages using otoliths accumulating in modern and fossil coral reef sediments. We present a reference collection which we use to identify otoliths found in reef sediments, showing that sediment otolith assemblages are on the whole representative of the native benthic and pelagic fish communities living on reefs. The relatively rare occurrences of some non-reef taxa support the general conclusion that predation is the most probable route by which otoliths accumulate in reef sediments. Preservation of otoliths in the Caribbean reef sediments we studied was generally good and the attainable taxonomic resolution was limited by the majority of otoliths originating from juvenile fishes rather than preservation state. Radiocarbon analysis conducted directly on representatively-small otoliths found in reef sediments in actively accreting branching coral framework show that time averaging is not excessive. In this habitat, the majority of otoliths in sea-floor sediments were modern in age, although this is highly unlikely to be the case in habitats where bioturbation is high.

We conclude that modern and fossil otolith assemblages in reef sediments can provide a powerful approach to reconstruct reef fish communities, establish pre-human baselines and explore the spatial and temporal dynamics of reef fish communities. These condensations of fish remains can provide a complementary approach to classic fish surveys which tend to provide “snapshots” of fish communities. Otolith assemblages, on the other hand, are accumulations over time that average-out temporal ecological fluctuations. Otoliths in the sediments have the benefit over other survey approaches by including fishes that are often missed in surveys, such as nocturnal and cryptobenthic fishes. The majority of reef sediment otolith assemblages are dominated by the cryptobenthic components, a result which is likely driven by the fact that such fishes are highly abundant, grow fast and have relatively short reproductive life histories.

A major constraint of this approach is the limited taxonomic resolution that is possible. Our improving reference collection will help go some way to advance this resolution. Nevertheless, it is likely that there will always be a substantially large proportion of reef sediment otoliths that cannot be identified to species because diversity is high and a large proportion of the otoliths are juveniles, making identification not only challenging but in many cases unresolvable. Nonetheless, in many cases, otoliths can be confidentially assigned to basic functional guilds on reefs, and this could represent an extremely powerful approach to explore the questions about trophic structure, energy flow and food web structure on reefs over space and time.

## Supporting information

S1 Fig **Facies, distribution of principal habitats (A) and stratigraphy (B) of mid-Holocene excavation sites in Bocas del Toro, western Panama.** Description of trenches is ordered roughly from the youngest to the oldest in (B).
(TIF)

S2 Fig Bulk sampling from sub-Recent branching coral reef framework on living reefs. (TIF)

S3 Fig Fish otoliths from the rotenone samplings in Bocas del Toro. Images are inner views and scale bars = 1 mm unless otherwise indicated.
(TIF)

S4 Fig Fish otoliths from the rotenone samplings in Bocas del Toro. Images are inner views and scale bars = 1 mm unless otherwise indicated.
(TIF)

S5 Fig Fish otoliths from the rotenone samplings in Bocas del Toro. Images are inner views and scale bars = 1 mm unless otherwise indicated.
(TIF)

S6 Fig Fish otoliths from the rotenone samplings in Bocas del Toro. Images are inner views and scale bars = 1 mm unless otherwise indicated.
(TIF)

S7 Fig Differences in preservation status of the otolith specimens according to the proportion of identifiable otoliths in sampled region and age. Samples from the same site are grouped. Box plot with lower (25th percentile), median and upper (75th percentile) boundaries, whiskers of 10th and 90th percentiles, and outliers (solid circle) outside of 10th and 90th percentiles are presented.
(TIF)

S1 Table Fish otolith information with corresponding radiocarbon (^14^C) measurement data from each specimen that was used to determine a date of formation. Measurements were correlated in time by alignment to a regional coral ^14^C reference record (Fig 13). There were two potential dates of formation for measurements that crossed the coral reference, the bomb ^14^C rise (~1958–1970) and decline periods (post-1982). Two specimens fell outside these periods and were either pre-bomb (earlier than ~1958) or during the peak period (~1970–1982). Uncertainty for formation dates is approximately ± 2 years for the rise period and ± 5 years for the decline period based on 95% prediction intervals.
(XLSX)

S2 Table Summary of reference otolith collection at the Naos Marine Laboratories of the Smithsonian Tropical Research Institute (STRI). (XLSX)

S3 Table Average otolith density by site. SD = standard deviation.
(XLSX)

S4 Table Shannon’s diversity index, Simpson's diversity, and Pielou's evenness index by region and age. (XLS)

S5 Table Fish identified from the rotenone samplings in Bocas del Toro. (XLSX)

S1 Text Summary of statistics. (DOCX)

S2 Text Details of carbon dating methods. (DOCX)
