# Supplementary figures and images for: Reconstructing reef fish communities using fish otoliths in coral reef sediments
Source: PLoS One. 2019 Jun 14;14(6):e0218413. doi: 10.1371/journal.pone.0218413 (PMC6568422; doi:10.1371/journal.pone.0218413)

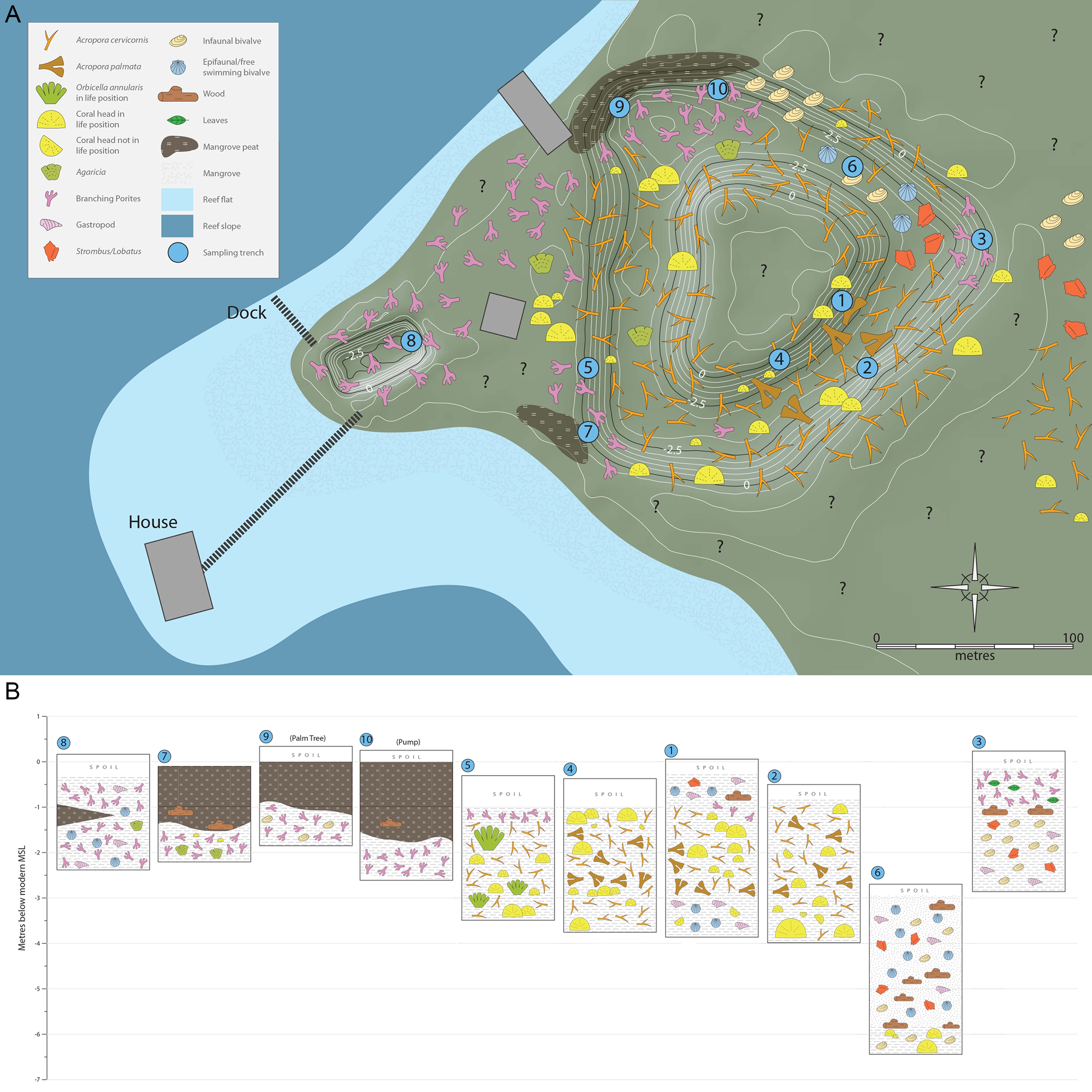

Supplement: S1 Fig — Facies, distribution of principal habitats (A) and stratigraphy (B) of mid-Holocene excavation sites in Bocas del Toro, western Panama. Description of trenches is ordered roughly from the youngest to the oldest in (B). (TIF) [file pone.0218413.s001.tif]

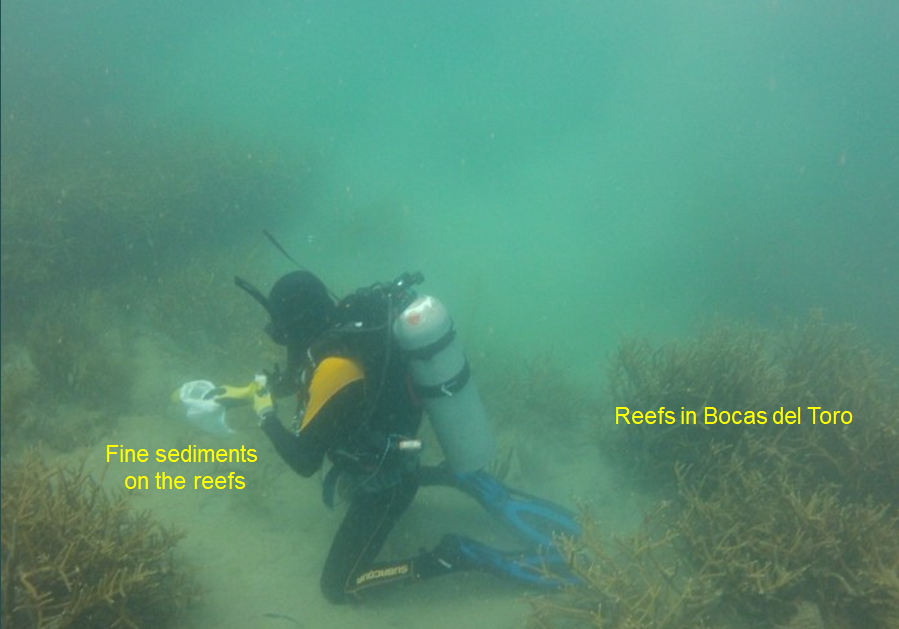

Supplement: S2 Fig — (TIF) [file pone.0218413.s002.tif]

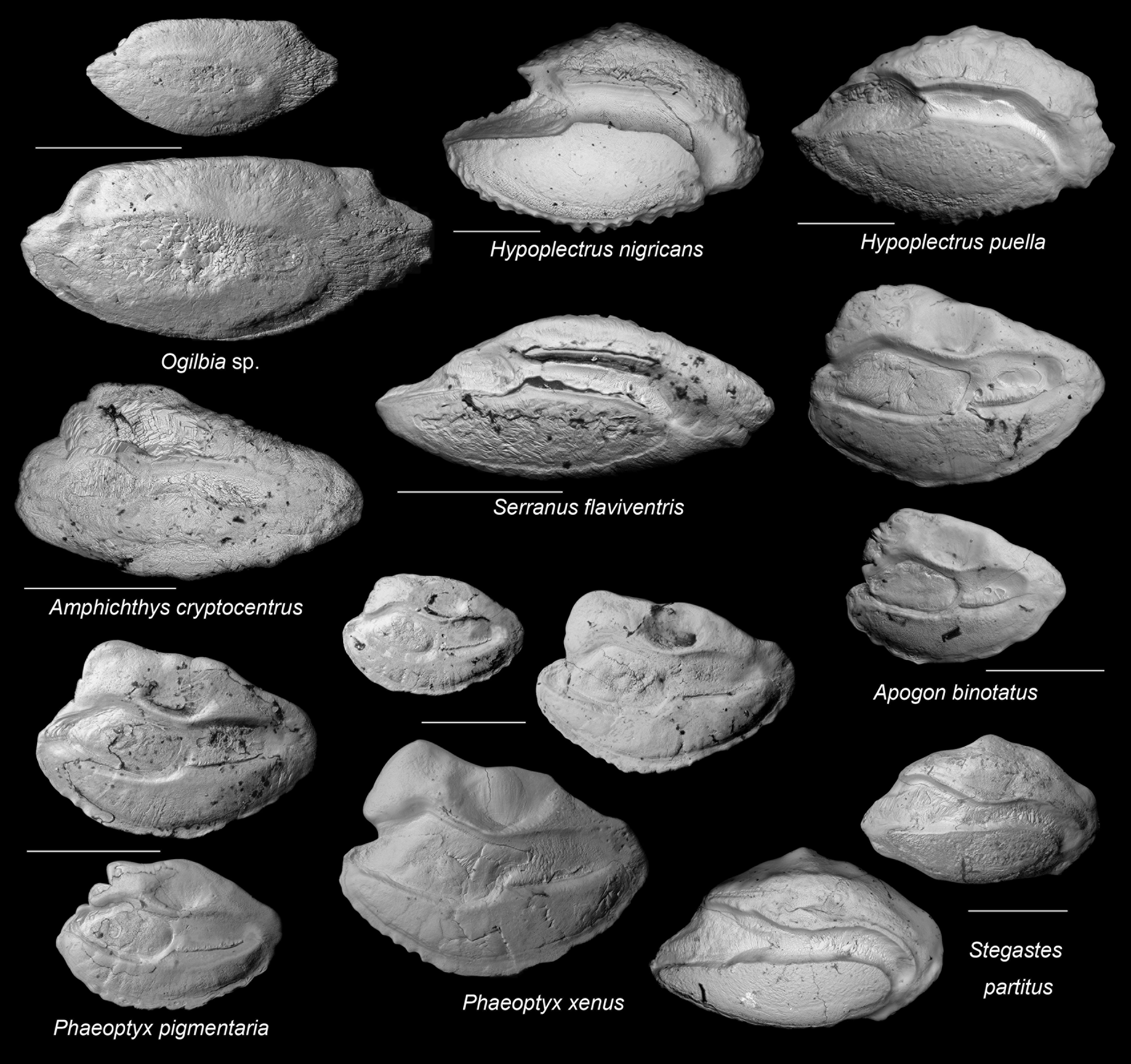

Supplement: S3 Fig — Images are inner views and scale bars = 1 mm unless otherwise indicated. (TIF) [file pone.0218413.s003.tif]

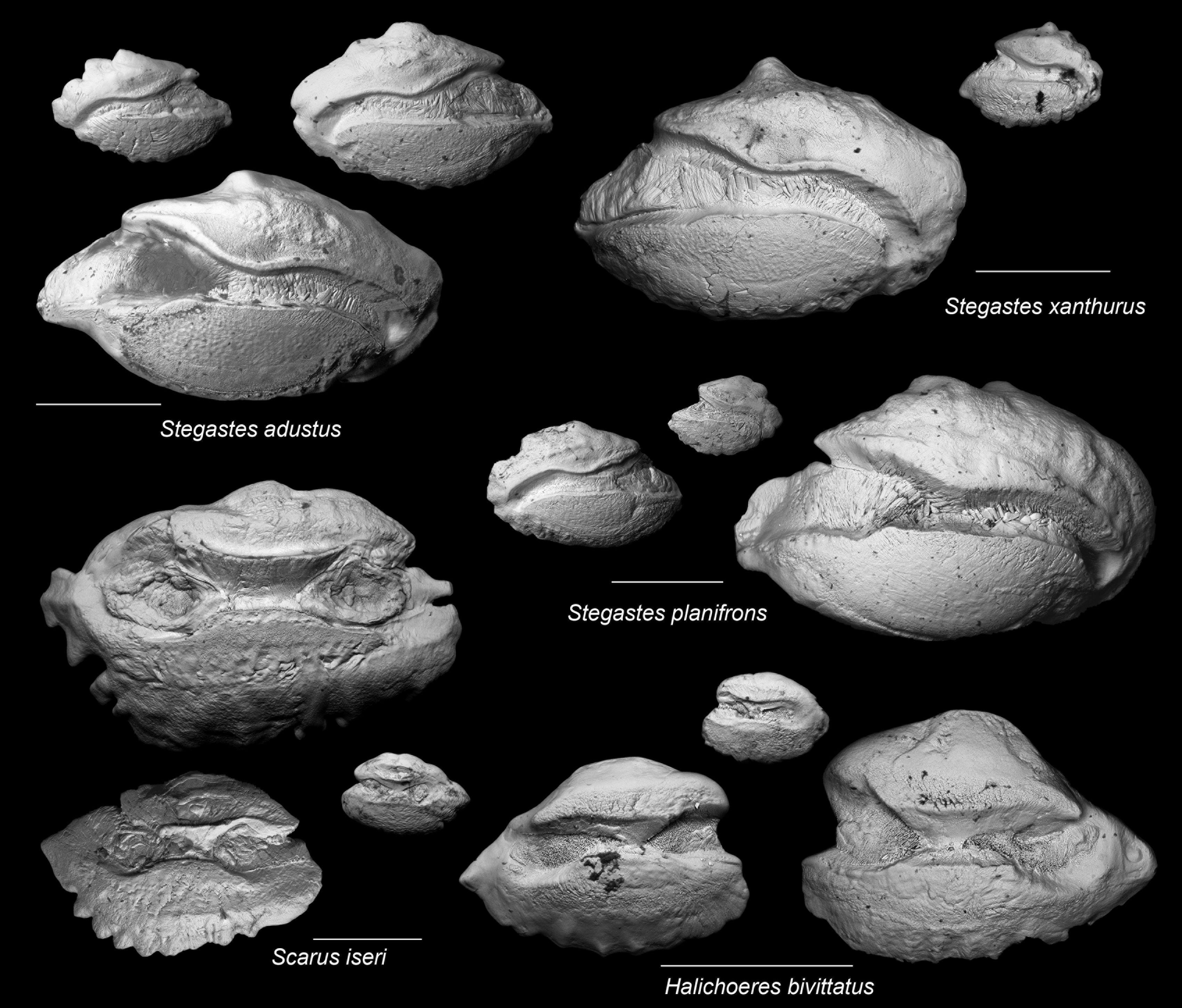

Supplement: S4 Fig — Images are inner views and scale bars = 1 mm unless otherwise indicated. (TIF) [file pone.0218413.s004.tif]

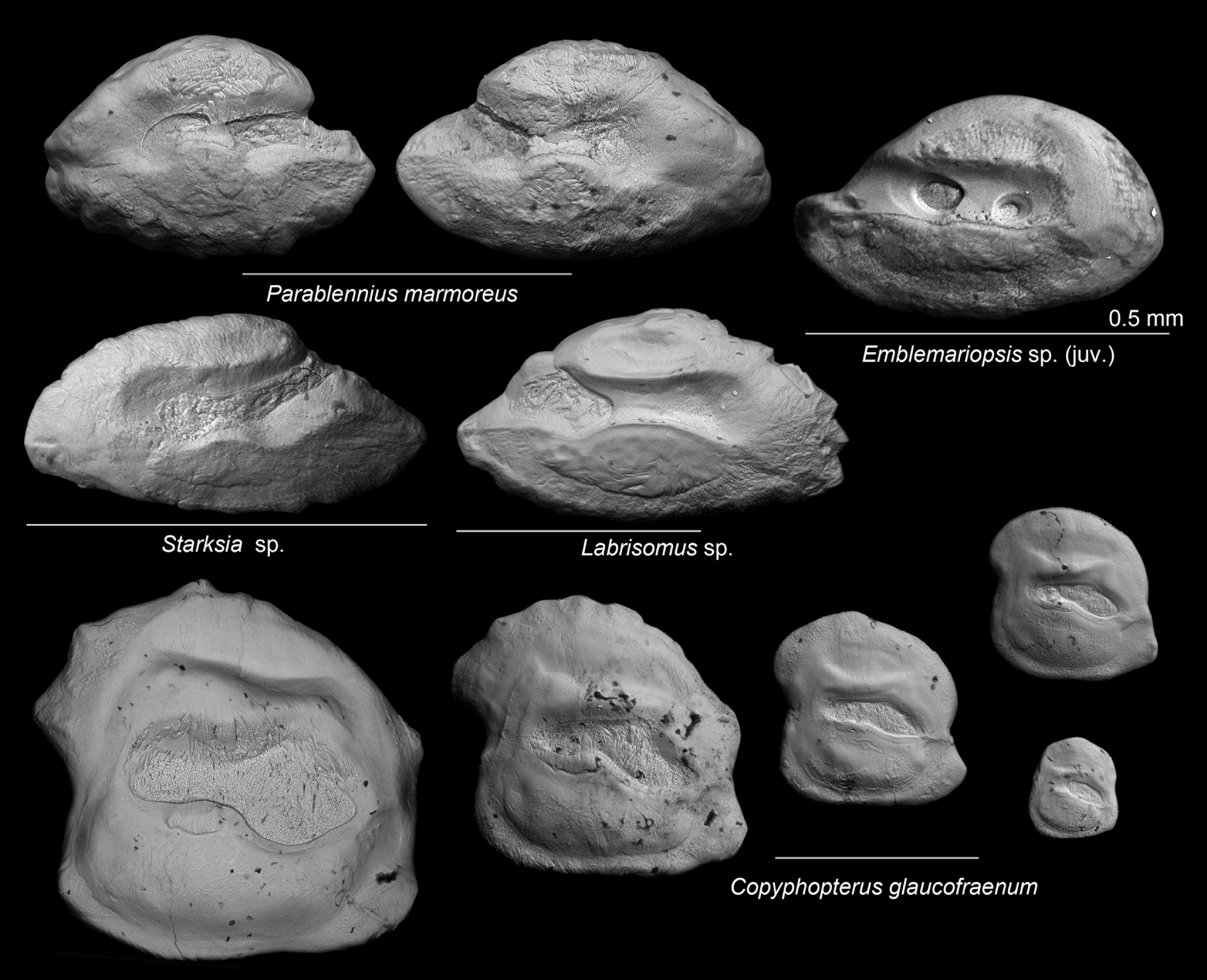

Supplement: S5 Fig — Images are inner views and scale bars = 1 mm unless otherwise indicated. (TIF) [file pone.0218413.s005.tif]

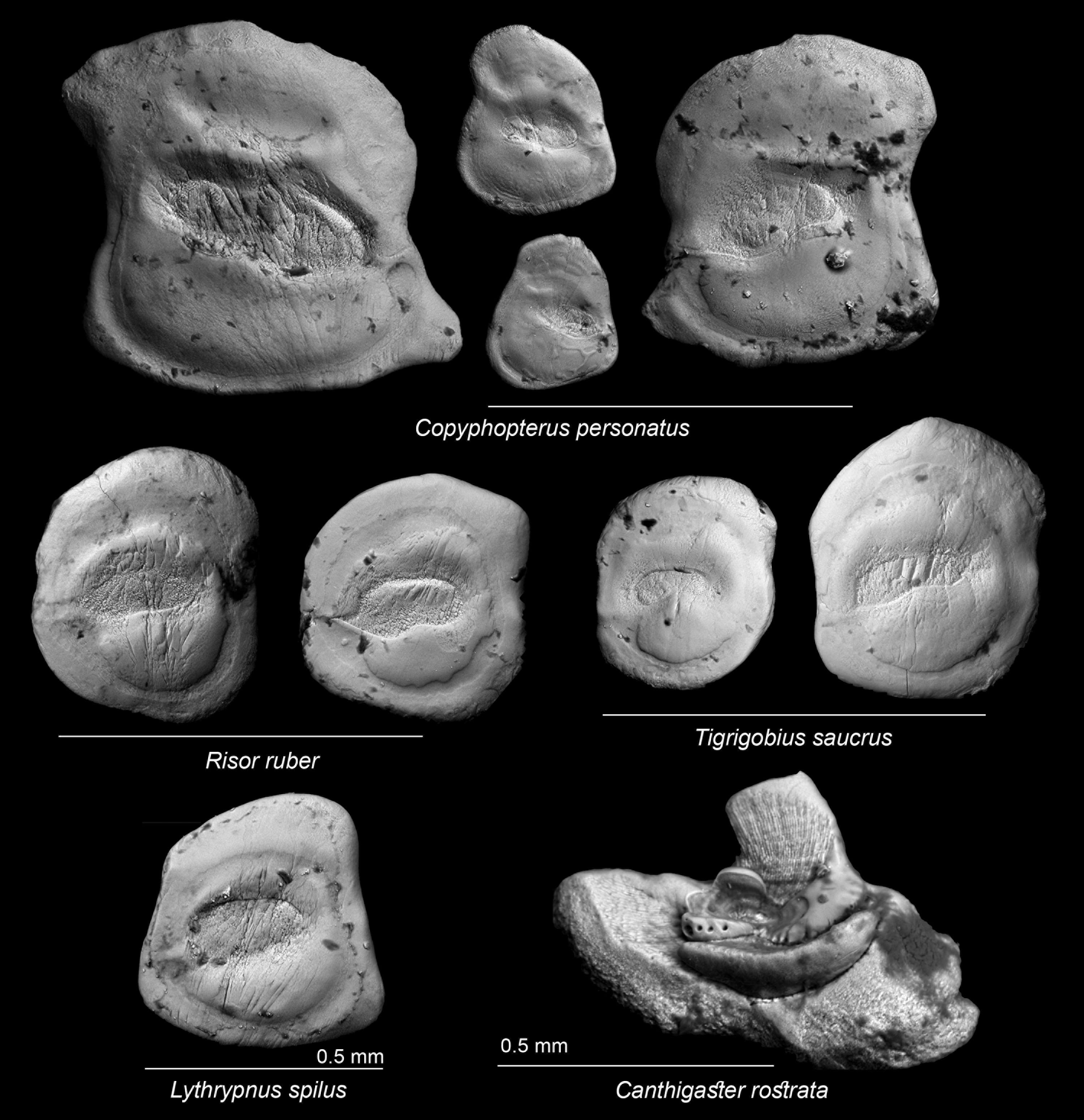

Supplement: S6 Fig — Images are inner views and scale bars = 1 mm unless otherwise indicated. (TIF) [file pone.0218413.s006.tif]

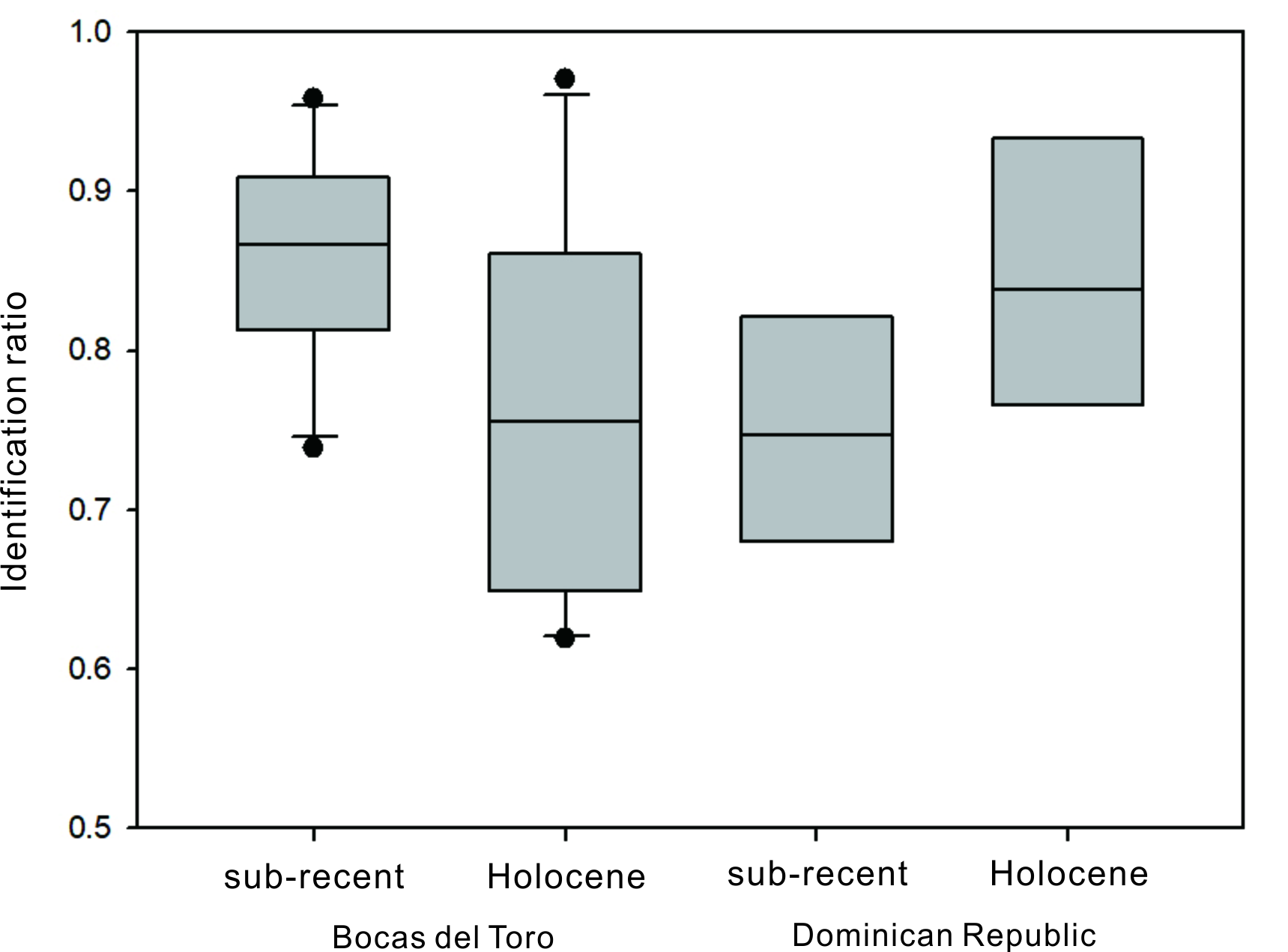

Supplement: S7 Fig — Samples from the same site are grouped. Box plot with lower (25th percentile), median and upper (75th percentile) boundaries, whiskers of 10th and 90th percentiles, and outliers (solid circle) outside of 10th and 90th percentiles are presented. (TIF) [file pone.0218413.s007.tif]
