# Supplementary material for: Reconstructing reef fish communities using fish otoliths in coral reef sediments
Source: PLoS One. 2019 Jun 14;14(6):e0218413. doi: 10.1371/journal.pone.0218413 (PMC6568422; doi:10.1371/journal.pone.0218413)
Supplement: S1 Text — (DOCX) [file pone.0218413.s013.docx]

# Summary of statistics

Data were tested for homogeneity of variance and normality. Shapiro-Wilk tests found that data from Bocas del Toro were not normally distributed (W = 0.84, p < 0.01). When Bocas del Toro data were separated by time, it was found that sub-recent data were normal (W = 0.9369, p > 0.05.) whereas Holocene data were not (W = 0.6483, p < 0.01). Both sub-recent (W = 0.8863, p > 0.05) and Holocene data (W = 0.8885, p > 0.05) from the Dominican Republic were found to be normally distributed. The variance was also found to be unequal between Bocas del Toro and the Dominican Republic with an F-test (p = 0.000014). A Mann-Whitney U test revealed that the average otolith density in Bocas del Toro (Mdn = 39.6) was not significantly higher than in the Dominican Republic (Mdn = 21.9) (Mann-Whitney U = 76, p = 0.1556). However, a T-test where the equal variance was not assumed did find the density differences to be significant (t(21) = 2.15, p = 0.0431). The two-tailed T-test has greater power, however, it also runs a higher chance of finding statistical significance when none is present. For that reason, and for the partial abnormality of our data distribution, we chose to report Mann-Whitney U in the main paper text. Standard deviations of densities can be found in S3 Table.
