# Supplementary material for: Reconstructing reef fish communities using fish otoliths in coral reef sediments
Source: PLoS One. 2019 Jun 14;14(6):e0218413. doi: 10.1371/journal.pone.0218413 (PMC6568422; doi:10.1371/journal.pone.0218413)
Supplement: S2 Text — (DOCX) [file pone.0218413.s014.docx]

# Details of carbon dating methods

To calculate the local bomb pulse ^14^C curve, we used the nearest bomb reference records for mixed surface waters of the Caribbean Sea taken from hermatypic coral cores off Puerto Rico and Venezuela [[1,2]](https://paperpile.com/c/JnCMJW/Lv8Q8+zwytO). Both corals were from the eastern end of the Caribbean Sea, but were most applicable because the broad Caribbean Current supplies surface waters to the Panama coast. The Puerto Rico record was from the southern coast of the island and the two records off Venezuela were from coral reefs well off shore in the Cariaco Basin (Isla Tortuga and Boca de Medio). Hence, the two northern and southern Caribbean locations would likely provide an indication of what the bomb ^14^C signal was for the mixed waters of the Caribbean Sea farther to the west, as the current enters an eddy off Panama in the Columbia Basin [[3]](https://paperpile.com/c/JnCMJW/9QxY5). Other coral records farther north, including Belize, reveal a more modern and elevated bomb ^14^C pattern that is representative of tropical waters in the Gulf of Mexico and western North Atlantic Ocean [[1,4]](https://paperpile.com/c/JnCMJW/Lv8Q8+4UkEF). Hence, the only applicable bomb ^14^C reference records for the coastal waters of Panama were from the two selected locations. These three coral records were combined and fitted with a Loess curve (spline interpolation smoothing parameter = 0.3, 2-parameter polynomial; Sigmaplot 11.2) to delineate the central tendency of the bomb ^14^C signal (Fig 13).

Alignment of the measured otolith ^14^C values to calculate a year of formation was based on a linear regression of the coral ^14^C values covering the span of measured otolith F^14^C values. This encompassed the mid to upper bomb ^14^C rise period and most of the decline period. Uncertainty in the years of formation was estimated from the 95% prediction intervals of the linear regression to capture the full variance range. Bomb ^14^C peak values were relegated to greater uncertainty because of the >10-year span of variable ^14^C levels across the plateau (after the rise and before the decline, ~1970–1982). Alignment of pre-bomb ^14^C levels was determined from true ^14^C dating and a marine reference record [[5]](https://paperpile.com/c/JnCMJW/QtymP). Calibrated calendar age was determined using the ΔR value determined from the Boca de Medio location because of its position in the Caribbean Current (ΔR = -31 ± 9 yr) [[2]](https://paperpile.com/c/JnCMJW/zwytO) and was used for radiocarbon age correction in CALIB Rev7.1.0 (http://calib.org/calib/calib.html) using the Marine13 ^14^C record [[5]](https://paperpile.com/c/JnCMJW/QtymP).

# References

1. [Kilbourne KH, Quinn TM, Guilderson TP, Webb RS, Taylor FW. Decadal- to interannual-scale source water variations in the Caribbean Sea recorded by Puerto Rican coral radiocarbon. Clim Dyn. 2007; 29: 51–62.](http://paperpile.com/b/JnCMJW/Lv8Q8)

2. [Wagner AJ, Guilderson TP, Slowey NC, Cole JE. Pre-Bomb Surface Water Radiocarbon of the Gulf of Mexico and Caribbean as Recorded in Hermatypic Corals. Radiocarbon. Cambridge University Press; 2009; 51: 947–954.](http://paperpile.com/b/JnCMJW/zwytO)

3. [Richardson PL. Caribbean Current and eddies as observed by surface drifters. Deep Sea Res Part 2 Top Stud Oceanogr. 2005; 52: 429–463.](http://paperpile.com/b/JnCMJW/9QxY5)

4. [Andrews AH, Barnett BK, Allman RJ, Moyer RP, Trowbridge HD. Great longevity of speckled hind (*Epinephelus drummondhayi*), a deep-water grouper, with novel use of postbomb radiocarbon dating in the Gulf of Mexico. Can J Fish Aquat Sci. 2013; 70: 1131–1140.](http://paperpile.com/b/JnCMJW/4UkEF)

5. [Reimer PJ, Bard E, Bayliss A, Warren Beck J, Blackwell PG, Ramsey CB, et al. IntCal13 and Marine13 Radiocarbon Age Calibration Curves 0–50,000 Years cal BP. Radiocarbon. Cambridge University Press; 2013; 55: 1869–1887.](http://paperpile.com/b/JnCMJW/QtymP)
